# Prenatal glucocorticoid exposure selectively impairs neuroligin 1-dependent neurogenesis by suppressing astrocytic FGF2–neuronal FGFR1 axis

**DOI:** 10.1007/s00018-022-04313-2

**Published:** 2022-05-13

**Authors:** Gee Euhn Choi, Chang Woo Chae, Mo Ran Park, Jee Hyeon Yoon, Young Hyun Jung, Hyun Jik Lee, Ho Jae Han

**Affiliations:** 1 Department of Veterinary Physiology, College of Veterinary Medicine, Research Institute for Veterinary Science, and BK21 Four Future Veterinary Medicine Leading Education and Research Center, Seoul National University, Seoul, 08826 Korea; 2 Laboratory of Veterinary Physiology, College of Veterinary Medicine, Chungbuk National University, Cheongju, 28644 Chungbuk Korea; 3 Institute for Stem Cell and Regenerative Medicine (ISCRM), Chungbuk National University, Cheongju, 28644 Chungbuk Korea

**Keywords:** Prenatal stress, Glutamatergic synaptogenesis, Astrocyte–Neuron interaction, Neural development

## Abstract

Exposure to maternal stress irreversibly impairs neurogenesis of offspring by inducing life-long effects on interaction between neurons and glia under raging differentiation process, culminating in cognitive and neuropsychiatric abnormalities in adulthood. We identified that prenatal exposure to stress-responsive hormone glucocorticoid impaired neurogenesis and induced abnormal behaviors in ICR mice. Then, we used human induced pluripotent stem cell (iPSC)-derived neural stem cell (NSC) to investigate how neurogenesis deficits occur. Following glucocorticoid treatment, NSC-derived astrocytes were found to be A1-like neurotoxic astrocytes. Moreover, cortisol-treated astrocytic conditioned media (ACM) then specifically downregulated AMPA receptor-mediated glutamatergic synaptic formation and transmission in differentiating neurons, by inhibiting localization of ionotropic glutamate receptor (GluR)1/2 into synapses. We then revealed that downregulated astrocytic fibroblast growth factor 2 (FGF2) and nuclear fibroblast growth factor receptor 1 (FGFR1) of neurons are key pathogenic factors for reducing glutamatergic synaptogenesis. We further confirmed that cortisol-treated ACM specifically decreased the binding of neuronal FGFR1 to the synaptogenic *NLGN1* promoter, but this was reversed by FGFR1 restoration. Upregulation of neuroligin 1, which is important in scaffolding GluR1/2 into the postsynaptic compartment, eventually normalized glutamatergic synaptogenesis and subsequent neurogenesis. Moreover, pretreatment of FGF2 elevated neuroligin 1 expression and trafficking of GluR1/2 into the postsynaptic compartment of mice exposed to prenatal corticosterone, improving spatial memory and depression/anxiety-like behaviors. In conclusion, we identified neuroligin 1 restoration by astrocytic FGF2 and its downstream neuronal nuclear FGFR1 as a critical target for preventing prenatal stress-induced dysfunction in glutamatergic synaptogenesis, which recovered both neurogenesis and hippocampal-related behaviors.

**Supplementary Information:**

The online version contains supplementary material available at 10.1007/s00018-022-04313-2.

## Introduction

Stress is a high-risk factor for suppressing the development and differentiation of both embryonic and adult neural stem cells (NSC). While the effect of stress during adulthood on neural tissue is usually reversible, prenatal or early-postnatal stress continues to adversely affect neurogenesis throughout life by permanently downregulating neurogenic or neurotrophic factors [1–3]. As a result, both rodents and primates that have experienced prenatal stress often develop neuropsychiatric-like or cognitive deficits as juveniles or adolescents even if the stress has not been continued [4–6]. Glucocorticoid largely mediates these deleterious effects of prenatal stress and is most influential in the hippocampus, the center for emotion and memory formation of both in animals and humans due to its abundance of glucocorticoid receptor (GR) [7]. However, the transcription of GR differs depending on the maturity of neurons in that GR is not transiently expressed at a certain stage of immature neurons. Therefore, other glucocorticoid-mediated crosstalk pathways that are independent from GR can largely affect neural differentiation during early life [2, 8]. Furthermore, prenatal stress evokes significant changes to epigenetic profiles through DNA methylation/demethylation or chromatin remodeling, which last throughout life [9, 10]. These specific changes due to prenatal stress suggest that they are unique to fetal or infant brains, in which neurogenesis and gliosis rigorously occur. However, little is known about the underlying molecular and cellular alterations.

In adults, neuroprogenitor cells (NPCs), which occupy small portions of neuronal cells and are mostly derived from the subgranular zone (SGZ) of the hippocampus, undergo gliosis to buffer stress responses by triggering neurogenesis and strengthening brain circuits [11]. Astrocytes, the most abundant glial cell type, interact with neurons, especially by neurotrophic support to maintain CNS health during stress by aiding neurogenesis, synapse formation, and neural tissue repair [12, 13]. However, large portions of NPCs can be extensively affected by the damaging effect on astrocytes exposed to prenatal stress. Neurotrophic support by astrocytes is a determining factor in outcome of mid-to-late-stage prenatal neurogenesis in rodents and humans. For example, gliosis peaks during the second week of rodent fetal life, when the levels of several neurotrophic factors such as FGF2 and BDNF are also known to peak to aid synaptogenesis [14, 15]. During this period, prenatal stress reduces the astrocytic pool and changes the profiles of secreted factors, all of which contribute to permanent defects in neurogenesis and hippocampus-related cognition [16]. Changes in astrocytic reactivity or proliferation by glucocorticoid during early life can be hypothesized to alter neurotrophic support to neurons. Then, impaired glutamatergic or GABAergic synapse formation would follow due to a decline in downstream factors participating in migration, proliferation, and synaptic trafficking. Therefore, we sought to identify the interactions between the altered neurotrophic factors produced by these affected astrocytes and the responsive synaptogenic regulators as putative modulators of neurogenesis under prenatal glucocorticoid.

We exposed ICR fetuses to prenatal glucocorticoid to assess its effect on neurogenesis and subsequent behavioral deficits. In addition, we used human iPSC-derived NSCs, a common in vitro model for observing neurogenesis, to investigate how glucocorticoid affects synaptic plasticity and the differentiation potential of NSCs. Using both in vivo and in vitro models, the study addresses the effect of glucocorticoid on astrocyte–neuron interactions and the ensuing dysfunction of synaptogenesis, resulting in deficits in neurogenesis or behavior.

## Materials and methods

### Materials

Fetal bovine serum (#SH30088.031R) and antibiotic–antimycotic mixture (#15240062) were purchased from Hyclone (Logan, UT, USA) and Gibco (Grand Island, NY, USA), respectively. The antibodies of lamin A/C (#sc-20681), pancadherin (#sc-59876), gephyrin (#sc-25311), and β-actin (#sc-47778) were purchased from Santa Cruz Biotechnology (Paso Robles, CA, USA). The antibodies of MAP2 (#ab11267), GFAP (#ab7260), ALDH1L1 (aldehyde dehydrogenase 1 family member L1, #ab190298), STAT3 (#ab76318), GluR2 (#ab133477), NR1 (#ab109182), synaptophysin (#ab32127), and neuroligin 1 (#ab186279) were obtained from Abcam (Cambridge, MA, USA). The GR antibody (#12041S) was acquired from Cell Signaling Technology, Inc. (Danvers, MA, USA). The antibodies of vGAT (#NBP2-20857) and C3 (#NBP1-32080) were purchased from Novus Biologicals (Littleton, CO, USA). The antibodies of DCX (doublecortin, #PA5-17428), NeuN (#MA5-33103), c-Fos (#MA5-15055), vGLUT1 (#48–2400), GluR1 (#PA1-46,151), NR2B (#71–8600), FGF2 (#OSG00015W), FGFR1 (#MA1-26256), and PSD95 (#51–6900) were purchased from Thermo Fisher (Rockford, IL, USA). Cortisol (#H4001), corticosterone (#C2505), actinomycin D (#A1410), AMPA (α-amino-3-hydroxy-5-methyl-4-isoxazole propionic acid, #A6816), NMDA (N-methyl-D-aspartate, #M3262), DAPI (#23397), α-tubulin antibody (#T6074), and PSD95 antibody (#MAB1596) were obtained from Sigma Chemical Company (St. Louis, MO, USA). The plasmids for pcDNA3.1/FGFR1-c-eGFP, pcDNA3.1/NLGN1-c-eGFP, and pcDNA3.1/c-eGFP were purchased from KomaBiotech (Seoul, Korea).

### Cell culture

The NSC induction was conducted from iPSCs obtained from Kangstem Biotech (Seoul, Korea) using a neural induction medium (#A1647801, Gibco). We plated NSCs on geltrex LDEV-Free (#A1413302, Thermo Fisher)-coated plates. For neural differentiation, NSCs were cultured in a neurobasal medium (#21103049, Gibco) with 2% supplement B27 (#17504044, Gibco) and 1% Glutamax (#35050061, Gibco). For astrocytic differentiation, NSCs were incubated in high-glucose DMEM (#SH30022.01, Hyclone) containing 1% N-2 supplement (#17502048, Gibco), 1% FBS, and 1% Glutamax (#35050–061, Gibco). After 5 days of astrocytic differentiation, astrocytes were cultured on neural differentiation media for 48 h with cortisol, and ACM were harvested.

Cultures of mouse primary hippocampal astrocytes were performed as described in the previous protocol [17]. Hippocampal astrocytes from postnatal day 1 (P1) mice were acquired in compliance and approval with the Institutional Animal Care and Use Committee of Seoul National University (SNU-190523–1-1). In brief, hippocampal astrocytes cultured on poly- _D_-lysine coated six-well plates were incubated in MEM (Hyclone) supplemented with glucose (0.6% wt/vol), 10% horse serum (vol/vol), and 1% antibiotic–antimycotic mixture.

### Transfection of plasmid DNA

Before treatment, neurons were incubated with a mixture of plasmid DNA, opti-MEM, and lipofectamine 3000 (Thermo Fisher). Lipid-based transfection method using lipofectamine 3000 was performed in differentiating neurons following the previous protocol [18]. After incubation, neurons were replaced with fresh neurobasal media containing 2% B27 supplement and 1% Glutamax to remove lipofectamine reagent.

### Transfection of small interfering RNA (siRNA) for gene silencing

Neurons were grown until approximately 60% confluency of the plate. Before ACM treatment, differentiating neurons were incubated with a mixture of 25 nM indicated siRNA and the turbofect transfection reagent (Thermo Fisher) for 24 h according to the manufacturer’s instructions. The siRNA specific for human *GR* was purchased from Bioneer Corporation (Daejeon, Korea). The siRNA specific for nontargeting (NT) was obtained from Dharmacon (Lafayette, CO, USA).

### Reverse-transcription PCR and real-time PCR

RNA samples were isolated using MiniBEST Universal RNA extraction kit (Takara, Otsu, Shinga, Japan). To acquire cDNA, reverse transcription was conducted using 1 μg of RNA for 1 h at 45 °C, followed by 5 min at 95 °C with a Maxime RT-PCR premix kit (Intron Biotechnology, Seongnam, Korea). Then, cDNA samples were amplified using Quanti NOVA SYBR Green PCR Kits (Qiagen, Hilden, Germany). Real-time quantification of RNA targets was performed with a Rotor-Gene 6000 real-time thermal cycling system (Corbett Research, NSW, Australia). Data were acquired during the extension step and then melting curve analysis was conducted to validate the specificity and identity of the PCR products. The data were normalized with the *ACTB* mRNA expression levels and the relative gene expression was analyzed using the delta–delta Ct method.

### RNA sequencing analysis

For the transcriptome profiling, bilateral hippocampi from littermates of mice that received vehicle or corticosterone were dissected manually at P1. For each RNA sample, the library construction was performed using the SENSE 3’ mRNA-Seq Library Prep Kit (Lexogen, Vienna, Austria) according to the manufacturer’s instructions. High-throughput sequencing was conducted as single-end 75 sequencings using NextSeq 500 (Illumina, San Diego, CA, USA). RNA sequencing reads were aligned to the UCSC hg19 genome build. Scatter plots were generated on differentially expressed genes more than 2 folds between the littermates of mice with vehicle and the littermates of mice with corticosterone at pregnancy.

### Western blot analysis

The samples were lysed with the EzRIPA buffer (#WSE-7420, ATTO, Tokyo, Japan) containing protease and phosphatase inhibitors. Cell debris was removed by centrifugation (13,000 × g at 4 °C, 30 min). Protein determination was done by a bicinchoninic acid quantification assay (Thermo Fisher). Equal amount of sample proteins (1–5 μg) were subjected to 8–12% SDS-PAGE and transferred to a polyvinylidene fluoride membrane. Then, the membranes were blocked with 5% bovine serum albumin (Sigma Chemical Company) or 5% skim milk (Gibco) in tris-buffered saline containing 0.2% Tween-20 {TBST; 150 mM NaCl, 10 mM Tris–HCl (pH 7.6), 0.1% Tween-20} solution for 30 min. After washing with TBST three times, the membranes were probed with the primary antibody at 4 °C overnight. Next, the membranes were washed 3 times with TBST, followed by incubation with the HRP-conjugated secondary antibodies capturing mouse or rabbit IgG (Thermo Fisher) at room temperature for 2 h. The protein complexes were detected with chemiluminescence solution (BioRad, Hercules, CA, USA) and the densitometry analysis for quantification was performed using Image J software (developed by Wayne Rasband, National Institutes of Health, Bethesda, MD, USA).

### Co-immunoprecipitation

Primary antibodies were immobilized with SureBeads™ protein G magnetic beads (BioRad). Immobilized magnetic beads were incubated with the total lysates of cells (200 μg) at 4 °C overnight. Magnetic beads were pulled down by a magnet and then acquired. The antibody-bound protein was collected by incubation in elution buffer (Thermo Fisher). Protein analysis was performed by western blot where anti-mouse or rabbit IgG antibody was used as a negative control.

### Immunocytochemistry

Cultured cells were fixed with 4% paraformaldehyde for 15 min at room temperature and then incubated in 0.1% Triton X for 5 min. Cells were placed in 5% normal goat serum (NGS) in PBS for 1 h. Next, the cells were incubated overnight at 4 °C with primary antibody dissolved in 5% NGS. The primary antibodies used in immunocytochemistry were listed as follows: GluR1 (#PA1-46151, Thermo Fisher), GluR2 (#ab133477, Abcam), PSD95 (#MAB1596, Sigma Chemical Company), FGFR1 (#MA1-26256, Thermo Fisher), gephyrin (#sc-25311, Santa Cruz Biotechnology), MAP2 (#ab11267, Abcam), GFAP (#ab7260, Abcam), vGAT (#NBP2-20857, Novus Biologicals), NR1 (#ab109182, Abcam), NR2B (#71–8600, Thermo Fisher), FGF2 (#OSG00015W, Thermo Fisher), and vGLUT1 (#48–2400, Thermo Fisher). After being washed with PBS, the cells were applied for 2 h at room temperature with Alexa Fluor™ secondary antibody (Thermo Fisher) as appropriate. Images were acquired by super-resolution radial fluctuations (SRRF) imaging system (Andor Technology, Belfast, UK). The fluorescent intensity analysis and co-localization analysis with Pearson’s correlation coefficient were performed with Fiji software (developed by Wayne Rasband, National Institutes of Health, Bethesda, MD, USA).

### Fluorescence imaging of synaptic vesicle recycling

For measuring recycled synaptic vesicles, neurons were loaded with FM4-64 dye (#T3166, Thermo Fisher) and stimulated with high K^+^-solution, following the previous protocol [19]. Images were acquired before and after destaining with high K^+^-solution for 90 s at 1 s intervals using Eclipse Ts2™ fluorescence microscopy (Nikon, Tokyo, Japan) and underwent analysis of destaining kinetics.

### Flow cytometry

Cells and hippocampal tissues were collected and dissociated with Versene solution (Gibco). After being washed with PBS, cells were fixed with TF Fix/Perm buffer (#562575, BD Bioscience, Franklin Lakes, NJ, USA) and suspended in Perm/Wash buffer at 5 × 10^5^ cells per 100 μl. The cells were incubated with primary antibody at 4 °C for 2 h and then applied in secondary antibody at 4 °C for 1 h as appropriate. After washing with PBS, the samples were detected with flow cytometry (Quanta SC; Beckman Coulter, Brea, CA, USA), and data analysis was conducted with CytExpert 2.3 software provided by Beckman Coulter.

### Annexin V apoptosis assay

Annexin V-FITC/propidium iodide (PI) staining was conducted with an annexin V-FITC apoptosis detection kit (#BD 556547, BD Bioscience). After treatment, astrocytes were suspended in a binding buffer. Next, annexin V-FITC and PI were added to the samples and incubated for 15 min at room temperature. Apoptosis of the samples was detected with flow cytometry (Quanta SC) and data analysis was conducted with CytExpert 2.3. Annexin V-FITC only positive cells undergo early apoptosis, whereas PI-positive only cells undergo necrosis. Both annexin V-FITC and PI-positive cells undergo late apoptosis.

### BrdU labeling

For BrdU labeling, ICR mice received an intraperitoneal injection of 100 mg/kg BrdU (Boehringer Mannheim, Mannheim, Germany) 24 h before sacrifice. NSC-derived neurons were incubated with 10 μM BrdU (Thermo Fisher) for 45 min at 37 °C before staining for flow cytometry, following the manufacturer’s protocol.

### Chromatin immunoprecipitation (ChIP)

ChIP assay was conducted using an EZ-ChIP Chromatin Immunoprecipitation Kit (#17–371, EMD Millipore, Burlington, MA, USA) following the manufacturer’s instructions. Samples including protein-chromatin complexes were incubated with ChIP grade antibody for FGFR1, the RNA polymerase (RNAPol), and the normal IgG overnight at 4 °C. RNAPol and normal IgG were used as a positive and negative control, respectively. Sample DNA was acquired by supplied column and amplified by PCR using a designed primer. The sequences of *NLGN1* primer are as follows: forward primer, 5’-TGAAGCAAGCTCTTAAATGGTG-3’ and reverse primer, 5’-TAATCGTAACCCCAAAAGGAAA-3’. The sequences of *NLGN3* primer are as follows: forward primer, 5’-TGCTAAACAAATGGCAGGTG-3’ and reverse primer, 5’-ACCTCCACCTCAATCAGCAT-3’. The sequences of *NRXN2* primer are as follows: forward primer, 5’-CCAGCACCACTCTAACTGAAAC-3’ and reverse primer, 5’-GCTTTGCTGGGATAAAGACG-3’. The sequences of *NRXN3* primer are as follows: forward primer, 5’-TGCCTCAAGGGGTTTATTTTTA-3’ and reverse primer, 5’-ACACTGCGCCTTCTTTATCAC-3’. One percent of the sample chromatin extract was used as an input.

### Experimental design of animal study

Female pregnant ICR mice exposed to corticosterone mimic the maternal stress-induced mouse model since corticosterone is primarily responsive hormone to stress. Then, the hippocampus of littermates was mainly used for evaluating glucocorticoid effect on neurogenesis since the hippocampus is closely related to neuropsychiatric and cognitive function in the brain. Female pregnant ICR mice aged 8 weeks were used, in compliance and approval with the Institutional Animal Care and Use Committee of Seoul National University (SNU-200221-6-1). Both sexes of ICR mice aged 8 weeks were also used to compare the effect of glucocorticoid exposure between infant and adult mice, in compliance and approval with the Institutional Animal Care and Use Committee of Seoul National University (SNU-190917-6-1). Animals were kept under standard environmental conditions (22 °C relative humidity 70%; 12 h light: dark cycle; ad libitum access to food and drinking solution). At least six mice were utilized for each group throughout the study. Applying the size of samples (minimum of *n* = 3) can be acceptable if very low *p* values are observed rather than the large size of N including interfering results. Therefore, we set the minimum of *n* = 3 (western blotting, immunohistochemistry) and *n* = 5 (behavior test) to gain statistical power according to the previously published article of Brain [20]. The experiments were designed in compliance with the ARRIVE guidelines. Allocations of animals were randomly done to minimize the effects of subjective bias.

Corticosterone (10 mg/kg) was dissolved in the solution containing 50% propylene glycol in PBS and injected intraperitoneally, which induces maternal stress in female pregnant mice and adult stress in 8 week-old mice [13, 21]. Vehicle-treated mice were similarly injected with the solution containing propylene and PBS. The dosage and treatment period of FGF2 were modified from previous reports [14, 22]. FGF2 (20 ng/g) was dissolved in PBS and treated every 3 h 3 times at P1. FGF2 was injected subcutaneously into axillary space. Mice were monitored twice a day during all experiments.

### Y-maze spontaneous alternation test

Y-maze spontaneous alternation test depends on the innate instinct of rodents to differently explore new environments. Thus, this test is widely used for quantifying the spatial memory of rodents. Rodents often prefer to challenge a new arm of the Y-maze rather than returning back to the one previously explored. Before the performance, the animals were habituated to the testing room for 3 h to minimize the stress. Then, mice were allowed for 10 min to explore the *Y*-shaped maze purchased from Sam-Jung Company (Seoul, Korea) while the number of arm entries and triads was recorded to calculate the percentage of an alternation. Only an entry when all four limbs were within the arm was counted. The alternation value represents the number of alternations which was divided by the number of total triads, which equals the number of total entries-2.

### Forced swim test

Forced swim test is a behavior test for rodents to evaluate their depressive-like behavior. Before the performance, the animals were habituated to the testing room for 3 h to minimize the stress. Mice were subjected to a forced swim test for 6 min in a beaker (10 cm × 20 cm) filled with tap water at room temperature, and the trials were analyzed by Smart 3.0 video tracking system (developed by Panlab, Barcelona, Spain). Generally, only the last 4 min of the test is analyzed, because most mice rigorously try to escape the environment at the beginning of the test. Immobility was defined as floating or remaining motionless without leaning against the wall of the cylinder.

### Open field test

Open field test evaluates anxiety of rodents using their characteristics to explore the periphery of open field when anxious. Before the performance, the animals were habituated to the testing room for 3 h to minimize the stress. Mice were placed in the rectangular plastic black boxes (H30 × L30 × W30 cm) and activity was recorded for 10 min. Total distance explored and time spent in either center or periphery of the open field were analyzed using Smart 3.0 video tracking system.

### Dexamethasone suppression test and corticosterone assay

Before the test, the blood was acquired from the tail vein. For dexamethasone suppression test, mice were injected with dexamethasone (50 μl/kg in propylene glycol) 1 h before acquiring blood from abdominal aorta of mice just before sacrifice. Then, plasma was separated from whole blood by centrifugation. To analyze, the concentration of plasma corticosterone was measured using the corticosterone ELISA kit purchased from Enzo Life Science (Farmingdale, NY, USA), according to the manufacturer’s instructions.

### Synaptosome isolation

Synaptosome of mouse hippocampus and NSC-derived neurons was extracted using Syn-Per synaptic protein extraction reagent (Thermo Fisher). Hippocampus and neurons were homogenized with a Dounce grinder with 20 slow strokes. Then, the homogenates underwent centrifugation at 1200 × *g* for 10 min at 4 °C. After discarding the pellet, the supernatant was centrifuged at 15000 × *g* for 20 min at 4 °C. The supernatant and pellet represent the cytosol and synaptosome, respectively.

### Growth factor antibody array

The growth factors of ACM were analyzed by incubation with membranes of the RayBiotech C-Series Human Growth Factor Antibody Array C1 kit, AAH-GF-1 (RayBiotech, Norcross, GA, USA). The membranes were incubated in blocking buffer for 30 min and then placed with conditioned media overnight at 4 ℃. Membranes were then washed 5 times with wash buffer and incubated with biotin-conjugated antibodies for 2 h at room temperature. After 5 times of washing with wash buffer, the membranes were incubated with horseradish peroxidase-conjugated streptavidin for 2 h. After the washing process, the human growth factors were detected by enhanced chemiluminescence reagents using a chemiluminescence imaging system.

### ELISA for human and mouse FGF2

For the quantification of FGF2 in conditioned media or tissue lysates, the FGF2 ELISA assay was achieved with human FGF2 ELISA kit (Abcam, #ab246531) and mouse FGF2 ELISA kit (Abcam, #ab100670) according to supplier’s protocols.

### Immunohistochemistry (IHC)

Mice were subjected to deep anesthesia with zoletil (50 mg/kg) and perfused transcardially with calcium-free Tyrode’s solution, followed by 4% paraformaldehyde. The brains were post-fixed for 2 h in 4% paraformaldehyde and then dehydrated in 30% sucrose in PBS for 24 h at 4 °C. Serial transverse sections (40 μm) were conducted using a cryostat (Leica Biosystems, Nussloch, Germany). The brain tissues including dorsal or ventral hippocampus were fixed with 4% paraformaldehyde for 10 min, and then pre-blocked with 5% NGS in PBS at room temperature for 1 h. Brain samples were incubated with primary antibody overnight at 4 °C, followed by the secondary antibody for 2 h at room temperature as appropriate. The primary antibodies used in IHC were listed as follows: c-Fos (#MA5-15055, Thermo Fisher), DCX (#PA5-17,428, Thermo Fisher), NeuN (#MA5-33103, Thermo Fisher), GluR1 (#PA1-46151, Thermo Fisher), GluR2 (#ab133477, Abcam), PSD95 (#MAB1596, Sigma Chemical Company), FGFR1 (#MA1-26256, Thermo Fisher), and vGLUT1 (#48–2400, Thermo Fisher). All completed samples were visualized using SRRF imaging system. The fluorescent intensity analysis and determining Pearson’s correlation coefficient values were undertaken using Fiji software.

### Statistical analysis

Imaging experiments and animal tests were conducted and assessed in a blinded fashion. Sample sizes were kept similar between experimental groups and replicates of experiments. The sample size ‘*n*’ represents the number of biological independent replicates and statistical analyses were conducted using these independent values. The Shapiro–Wilk test was used to verify the normality of all data, which were then analyzed using parametric statistics. The unpaired student’s *t* test was performed to compare the means of the treatment groups with that of the control group. One-way ANOVA (with Dunnett’s multiple comparison test) or two-way ANOVA (with Tukey’s multiple comparison test) were used for analyzing the differences among multiple groups. For measuring co-localization levels in images, the values of Pearson’s correlation coefficient were obtained from images of each treatment group, and appropriate tests were applied to confirm whether changes were statistically significant. Results were expressed as mean value ± standard error of the mean (S.E.M.) and analyzed with the GraphPad Prism 6 software (Graphpad, CA, USA). A result with a *p* value of < 0.05 was considered statistically significant.

## Results

### Prenatal glucocorticoid exposure induces anxiety/depression-like behavior and spatial memory deficits by impairing neurogenesis

**Fig. 1:**
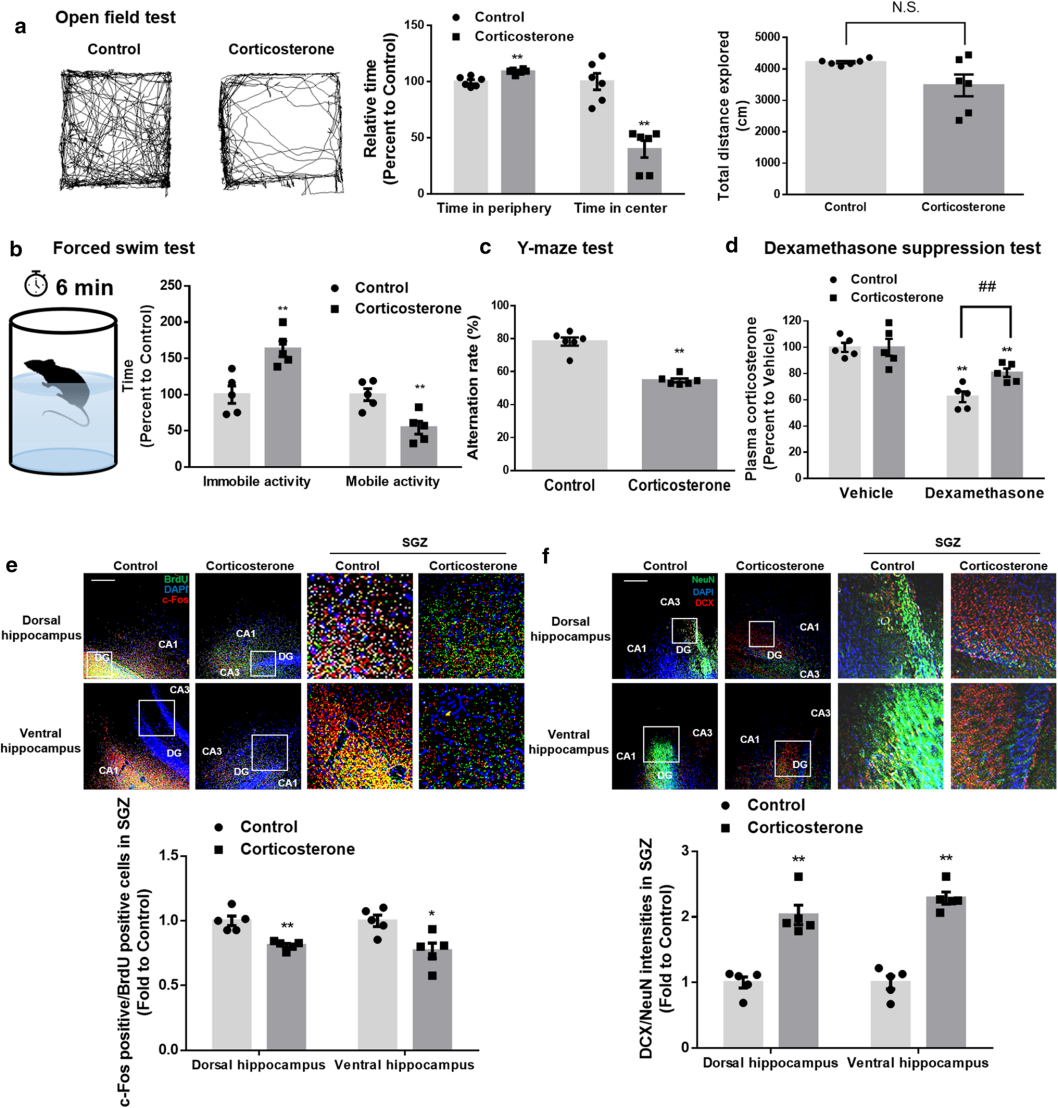
Prenatal corticosterone exposure triggers anxiety/depression-like behavior and spatial memory dysfunction by suppressing neurogenesis in mice. (**a**–**f**) Four weeks after exposure to maternal vehicle or corticosterone (10 mg/kg) at E14, mice underwent behavior tests, dexamethasone suppression test, and were sacrificed for IHC at P23. **a** Open field test was performed to assess anxiety-like behavior for 10 min. Relative time at the periphery/center and total distance explored were determined. Mice exposed to prenatal corticosterone were more active in the peripheral regions. *n* = 6. **b** Mice were presented to the forced swim test for 6 min. Immobile and mobile activities were determined during the last 4 min. Mice exposed to prenatal corticosterone showed more immobility. *n* = 5. **c** The mice were subjected to Y-maze test to evaluate spatial memory function. Mice exposed to prenatal corticosterone showed cognitive deficits. *n* = 6. **d** Before the dexamethasone suppression test, the blood was collected. Blood was extracted again 1 h after dexamethasone (50 μl/kg) was injected. Plasma corticosterone was determined by ELISA. Plasma corticosterone release was not well suppressed by dexamethasone in mice exposed to prenatal corticosterone. *n* = 5. ^*##*^ indicates *p* < *0.01* versus plasma corticosterone of dexamethasone-injected control mice. (**e**, **f**) Slide samples of both dorsal and ventral hippocampus for IHC were collected and the quantification analysis was performed in SGZ. The white square part of the images was enlarged. **e** For BrdU labeling, mice were injected intraperitoneally with BrdU (100 mg/kg) 24 h before sacrifice. The hippocampus was immunostained with BrdU (green), c-Fos (red), and DAPI (blue). The ratio of c-Fos- to BrdU-positive cells was decreased by prenatal corticosterone. Scale bars, 100 μm (magnification × 200). *n* = 5. **f** The hippocampus was immunostained with NeuN (green), DCX (red), and DAPI (blue). The ratio of DCX- to NeuN-positive cells was increased by prenatal corticosterone. Scale bars, 100 μm (magnification × 200). *n* = 5. All immunofluorescence images are representative. Two technical replicates from each animal (*n* = 5) were performed in results of IHC. Quantitative data are presented as a mean ± S.E.M. The representative images were acquired by SRRF imaging system. Two-sided unpaired student’s *t* test (Fig. 1a–c , e, f). Two-sided two-way ANOVA (Fig. 1d). ^*,**^indicates *p* < *0.05* and *p* < *0.01* versus control, respectively

We focused on the effect of stress on the hippocampus because of its critical role in processing emotionally salient information or regulating memory function [14]. Stress exposure releases glucocorticoid hormones, namely cortisol in humans and corticosterone in rodents. Previous studies have noted that excessive corticosterone exposure in fetal rodents triggers mood disorders or memory dysfunction during adulthood [4, 23]. Thus, we used about a 3 week-old mice model exposed to maternal corticosterone at embryonic day 14 (E14) to study the relationship between neurogenesis and emotional/memory behavior. Using an open field test to study anxiety behavior, in which mice with anxiety prefer to stay at the periphery, we found that those exposed to prenatal corticosterone were more active in the peripheral regions than in the center of the open field (Fig. 1a). To evaluate depression-like behavior, we performed a forced swim test, which determines immobility in a cylinder, an indicator of unwillingness to escape the environment. Mice exposed to prenatal corticosterone exhibited more immobility than the control mice (Fig. 1b). Furthermore, we assessed spatial memory function using the Y-maze, which uses the innate nature of rodents to explore new objects and observed that mice exposed to prenatal corticosterone exhibited dysfunctional spatial memory (Fig. 1c). Overall, maternal exposure to high levels of corticosterone influenced both neuropsychiatric and cognitive function in littermates. In adults, stress adaptation is usually well controlled by the hypothalamus–pituitary–adrenal (HPA) axis; however, exposure to stress during early life is known to permanently impair this axis. We thereby validated the hyperactivation of the HPA axis due to an impaired feedback mechanism using a dexamethasone suppression test during the dark cycle. Mice were treated with dexamethasone to suppress corticosterone release 1 h before serum acquisition. Dexamethasone effectively suppressed plasma corticosterone release in control mice, whereas mice exposed to prenatal corticosterone were more insensitive to the drug’s suppressive effects on the HPA axis (Fig. 1d). The effect of prenatal glucocorticoid exposure on neurogenesis was determined 28 days after initial exposure to prenatal corticosterone (P23) when the adult mice usually showed recovery. The dorsal and ventral hippocampus are widely known to be responsible for processing cognition and emotion, respectively [10]. Thus, we performed immunostaining in both regions and detected changes in neurogenesis, especially in the SGZ, where most hippocampal neurogenesis occurs [13]. First, we investigated the effect of corticosterone on neuronal activation. An immediate-early gene *c-fos* is activated after action potential firing, and this is used to evaluate activated neurons [24]. This gene is a proto-oncogene and found to be overexpressed in a variety of cancers. However, expression of c-Fos is widely used as an indirect marker of recent action potential in neurons in neural tissue due to its transient expression by stimulation [25]. The ratio of c-Fos-immunoreactive to surviving neurons binding to BrdU was decreased in the SGZ, suggesting that neural activity was reduced in both the dorsal and ventral hippocampus (Fig. 1e). Next, we determined neuronal maturation by staining neural tissue with DCX (immature neuron marker) and NeuN (marker for terminally differentiated neurons). Corticosterone exposure increased the ratio of DCX to NeuN fluorescence intensities in both the dorsal and ventral hippocampus, indicating that corticosterone suppressed neurogenesis (Fig. 1f). Together, these results demonstrated that exposure to prenatal corticosterone adversely affected neurogenesis which can impair mood/memory function.

### A1-like astrocytes induced by glucocorticoid suppress glutamatergic synaptogenesis

**Fig. 2:**
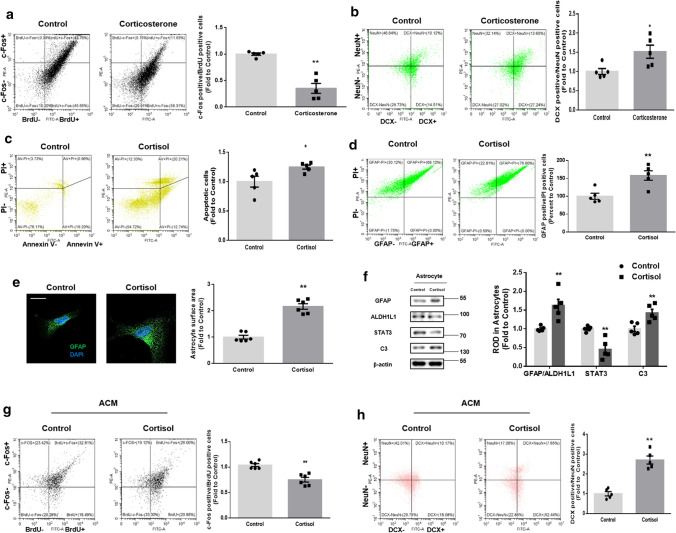
Glucocorticoid impairs neurogenesis through activating A1-like astrocytes. (**a**, **b**) After exposure to prenatal vehicle or corticosterone (10 mg/kg) at E14, the hippocampus from mice at P1 was collected. **a** For BrdU labeling, mice were injected intraperitoneally with BrdU (100 mg/kg) 18 h before sacrifice. Then, dissociated hippocampal cells were immunostained with BrdU (green) and c-Fos (red). The ratio of c-Fos-positive cells to BrdU-positive cells was analyzed by flow cytometry. The ratio of c-Fos- to BrdU-positive cells was decreased by prenatal corticosterone. *n* = 5. **b** The cells dissociated from the hippocampus were immunostained with DCX (green) and NeuN (red). The ratio of DCX-positive cells to NeuN-positive cells was analyzed by flow cytometry. The ratio of DCX- to NeuN-positive cells was increased by prenatal corticosterone. *n* = 5. (**c**, **d**) Human NSCs were incubated with astrocytic differentiation media with cortisol (1 μM) for 5 days. **c** The percentages of apoptotic cells (annexin V-positive cells) were analyzed by annexin V/PI analysis, measured by flow cytometry. Apoptotic cells were increased by cortisol. *n* = 5. **d** The differentiated cells were immunostained with GFAP (green) and PI (red). The ratio of GFAP-positive cells to PI-positive cells was analyzed by flow cytometry, which was increased by cortisol. *n* = 5. (**e**, **f**) Human NSCs were incubated with astrocytic differentiation media for 5 days and then cortisol (1 μM) was treated for 48 h. **e** Astrocytes were immunostained with GFAP (green) and DAPI (blue). Astrocytic surface area was analyzed by Image J, which was increased by cortisol. Scale bars, 20 μm (magnification × 1000). Three technical replicates for one dish were analyzed. Six biological replicates were performed. **f** The expressions of GFAP, ALDH1L1, STAT3, C3, and β-actin were detected by western blot. The levels of GFAP/ALDH1L1 and C3 were increased but STAT3 expression was reduced by cortisol. Five biological replicates were performed. (**g**, **h**) Human NSCs were incubated with astrocytic differentiation media for 5 days, and were then treated with cortisol (1 μM) for 48 h. Then, the ACM were collected and administered to the human NSCs incubated with neuronal differentiation media for 5 days. After 5 days of treatment, the neurons were immunostained as appropriate. **g** The cells were incubated with 10 μM BrdU (green) for 45 min at 37 °C and then immunostained with c-Fos (red). The ratio of c-Fos-positive cells to BrdU-positive cells was analyzed by flow cytometry, which was reduced by cortisol. *n* = 5. **h** The cells were immunostained with DCX (green) and NeuN (red). The ratio of DCX-positive cells to NeuN-positive cells was analyzed by flow cytometry, which was increased by cortisol. *n* = 5. All immunofluorescence images are representative. *n* = 5 from independent experiments with two technical replicates each. Quantitative data are presented as a mean ± S.E.M. The representative images were acquired by SRRF imaging system. Two-sided unpaired student’s *t* test was conducted. ^*,**^indicates *p* < *0.05* and *p* < *0.01* versus control, respectively

Mice exposed to stress during adulthood does not exhibit behavioral disorders 5 days after withdrawal of the stress [9]. However, we hypothesized that the effect of maternal glucocorticoid treatment on behavior deficits may persist even 4 weeks after glucocorticoid injection. Thus, we investigated whether changes in neurogenesis by glucocorticoid precede behavioral disorders. We injected corticosterone into pregnant mice at E14, when neuronal development and differentiation peak. 5 days after the injection when neurogenesis is phenotyped by stimulation (P1), it was then evaluated by flow cytometry. To assess whether corticosterone modified neuronal activity and neurogenesis, we performed immunostaining in the cells dissociated from the hippocampus at P1. Prenatal corticosterone injection reduced the ratio of c-Fos to BrdU immunoreactive cells by about 60% (Fig. 2a). Next, we observed that corticosterone exposure at E14 increased the ratio of DCX-positive to NeuN-positive neurons by about 50% (Fig. 2b). These data further demonstrated that corticosterone depleted the neurogenic potential of NPCs.

Growing evidence indicates that astrocytes can strongly regulate NSC dynamics during the fetal or infant stage through their secreted neurotrophic factors by enhancing angiogenesis, synapse formation, and the differentiation potential of neurons, motivating us to examine whether the astrocytic pool changes in response to glucocorticoid treatment [13, 26]. We treated cells with 1 μM cortisol or corticosterone, according to the stress-induced levels of glucocorticoid used in our previous research [21]. Then, we performed an annexin V apoptosis assay to detect the cell apoptosis. Approximately 20% more apoptosis (annexin V-positive cells) occurred in NSC-derived astrocytes exposed to cortisol for 5 days (Fig. 2c). These results concur with previous research demonstrating that glucocorticoid introduced at the fetal stage inhibits astrocyte proliferation and subsequent neurotrophic support, resulting in reduced synaptogenesis and neural development; however, contradictions to this suggestion also exist [12]. Astrocytes show heterogeneity in their reactions to various stimuli; a shift to A1-like reactive and inflammatory astrocytes, which lose their neurotrophic capacity, is induced in certain brain disorders or following damage [27]. In contrast, A2-like reactive and protective astrocytes aid neuronal growth and synapse repair to maintain brain homeostasis following stimulations such as ischemic injury [28–30]. To determine whether cortisol shifts astrocytes into reactive and pathological phenotypes, we first performed flow cytometry. We found that cortisol triggered reactive astrocytosis, because about 40% elevated levels of the reactive astrocyte marker GFAP compared with the nuclei marker PI were observed (Fig. 2d). Furthermore, cortisol increased the surface area of astrocytes, suggesting that they became reactive (Fig. 2e). We then distinguished which type of reactive astrocytes was dominant following exposure to cortisol. Western blotting revealed that cortisol elevated the ratio of the pan-reactive astrocytic marker GFAP to the astrocyte-specific marker ALDH1L1 (Fig. 2f). Simultaneously, cortisol decreased STAT3 but elevated C3 expression, which are the representative proteins for protective A2-like and neurotoxic A1-like astrocytic markers, respectively [30, 31]. We also confirmed that corticosterone elevated the ratio of GFAP to ALDH1L1 and the levels of C3, but decreased STAT3 levels in mouse primary hippocampal astrocytes (Supplementary Fig. 1). To observe the deleterious effects of A1-like astrocytes on neurogenesis, we extracted ACM treated with cortisol and administered them to differentiating neurons for 5 days. As shown in (Fig. 2g–h) cortisol-treated ACM reduced the neuronal activity (about 20% decreased ratio of c-Fos to BrdU-positive cells) and maturation of neurons (about threefold increase in the ratio of DCX to NeuN-positive cells). Collectively, these findings suggested that glucocorticoid critically depleted the neurogenic potential of NSCs by transforming astrocytes into A1-like reactive astrocytes which may not release neurotrophic factors.

**Fig. 3:**
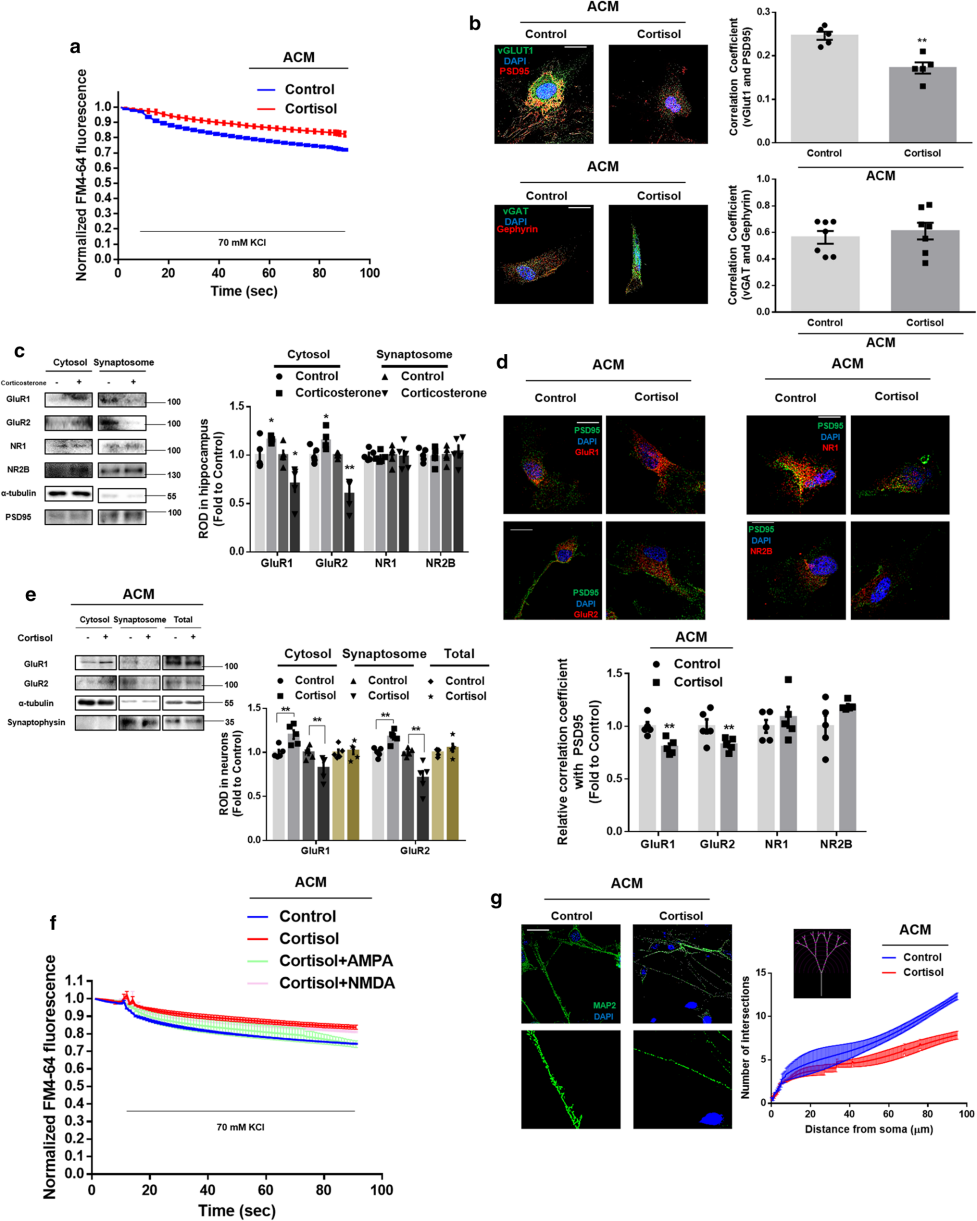
Glucocorticoid-induced change in astrocyte homeostasis inhibits glutamatergic synaptogenesis. (**a**, **b**, **d**–**g**) 5 days after astrocytic differentiation, human NSCs were treated with cortisol (1 μM) for 48 h and the ACM were collected. Human NSCs were cultured in neuronal differentiation media for 5 days and ACM were added for 48 h. **a** Conditioned neurons were stained with FM4-64 dye and stimulated with a high K^+^ buffer for destaining. Time-lapse imaging was performed over 90 s at 1 s intervals with an Eclipse Ts2™ fluorescence microscopy. Cortisol-treated ACM reduced synaptic vesicle recycling. *n* = 5. **b** (Upper images) The visualization of vGLUT1 (green), PSD95 (red), and DAPI (blue) was done in conditioned neurons. (Lower images) The detection of vGAT (green), gephyrin (red), and DAPI (blue) was performed in conditioned neurons. Pearson’s correlation coefficient between GFP and RFP fluorescence was quantified by using Fiji. Cortisol-treated ACM decreased co-localization between vGLUT1 and PSD95. Scale bars, 20 μm (magnification × 1000). *n* = 5 from independent plates for each experiment (glutamatergic and GABAergic synaptogenesis). **c** After exposure to prenatal vehicle or corticosterone (10 mg/kg) at E14, the hippocampus from mice at P1 was collected and underwent fraction to gain synaptosome. The expressions of GluR1/2, NR1, NR2B, α-tubulin, and PSD95 were detected by western blot. The α-tubulin and PSD95 were used as a loading control for cytosol and synaptosome, respectively. The levels of GluR1/2 at synaptosome were decreased by prenatal corticosterone. *n* = 5. **d** (Left) PSD95 (green), GluR1/2 (red), and DAPI (blue) were visualized through immunostaining. (Right) PSD95 (green), NR1 or NR2B (red), and DAPI (blue) were visualized through immunostaining. Scale bars, 20 μm (magnification × 1000). Pearson’s correlation coefficient between GFP and RFP fluorescence was quantified by using Fiji. Cortisol-treated ACM decreased co-localization between GluR1/2 and PSD95. *n* = 5. **e** The expressions of GluR1/2, α-tubulin, and synaptophysin in cytosol, synaptosome, and total lysates were detected by western blot. The α-tubulin and synaptophysin were used as a loading control for cytosol and synaptosome, respectively. The levels of GluR1/2 at synaptosome were decreased by cortisol-treated ACM. *n* = 5. **f** Before measuring the kinetics of FM4-64 dye, conditioned cells were pretreated with either AMPA (100 μM) or NMDA (100 μM). Then, neurons were stimulated with a high K^+^ buffer for destaining. Time-lapse imaging was performed over 90 s at 1 s intervals with an Eclipse Ts2™ fluorescence microscopy. Reduced synaptic vesicle recycling by cortisol-treated ACM was recovered by AMPA pretreatment. *n* = 5. **g** Conditioned neurons were immunostained with MAP2 (green) and DAPI (blue). The Sholl analysis was performed for analyzing neurite outgrowth. Scale bars, 50 μm (magnification × 400). Cortisol-treated ACM impaired neurite outgrowth. *n* = 5. All immunofluorescence images are representative. *n* = 5 from independent experiments with two technical replicates each. Quantitative data are presented as a mean ± S.E.M. The representative images were acquired by SRRF imaging system. Two-sided unpaired student’s *t* test (Fig. 3b–e). ^*,**^indicates *p* < *0.05* and *p* < *0.01* versus control, respectively

Reduced neurotrophic aid from astrocytes often culminates in less neuronal excitability, which usually precedes neurogenesis defects, due to the role of neurotrophic factors in glutamatergic or GABAergic synapse formation [32]. Thus, we measured the kinetics of FM4-64 destaining to validate the adverse effect of cortisol-treated ACM on the synaptic current in neurons [19]. When administered to differentiating neurons for 48 h, cortisol-treated ACM reduced the rate of FM4-64 dye release, indicating that synaptic vesicle recycling and synaptic current activity were reduced (Fig. 3a). To differentiate which types of synapse formation were prevented by cortisol-treated ACM, we stained the neurons with excitatory and inhibitory synaptic markers. As seen in (Fig. 3b), cortisol-treated ACM downregulated excitatory synapse formation (co-localization between presynaptic protein vGLUT1 and postsynaptic protein PSD95) but did not induce a significant change in inhibitory synapse formation (co-localization between presynaptic protein vGAT and postsynaptic protein Gephyrin). Furthermore, to determine which glutamatergic pathway was suppressed, synaptosome was extracted from P1 mice exposed to corticosterone at E14. As shown in (Fig. 3c), corticosterone reduced the levels of AMPA receptor (AMPAR) subtypes GluR1/2 in the synaptosome of the hippocampus whereas the NMDA receptor (NMDAR) subtypes NR1 and NR2B exhibited no significant level changes in each compartment. In line with the in vivo results, cortisol-treated ACM reduced the recruitment of GluR1/2 into synaptic membrane, whereas no significant transport of NR1 and NR2B was detected in the immunofluorescence results (Fig. 3d). Furthermore, cortisol-treated ACM reduced synaptic trafficking of GluR1/2 in human differentiating neurons, whereas their expression remained unchanged (Fig. 3e). To confirm whether AMPA-mediated glutamatergic transmission in neurons was impaired by cortisol-treated ACM, we showed that synaptic vesicle recycling was recovered by AMPA treatment, not by NMDA treatment (Fig. 3f). Furthermore, using Sholl analysis, the number of intersections was reduced by cortisol-treated ACM, meaning that neurite outgrowth and subsequent synaptogenesis were impaired (Fig. 3g). These data suggested that astrocytic phenotype changes caused by glucocorticoid highly affect AMPAR—but not NMDAR-mediated glutamatergic synaptogenesis of neurons [33].

### Glucocorticoid impairs communication between astrocytic FGF2 and neuronal FGFR1

**Fig. 4:**
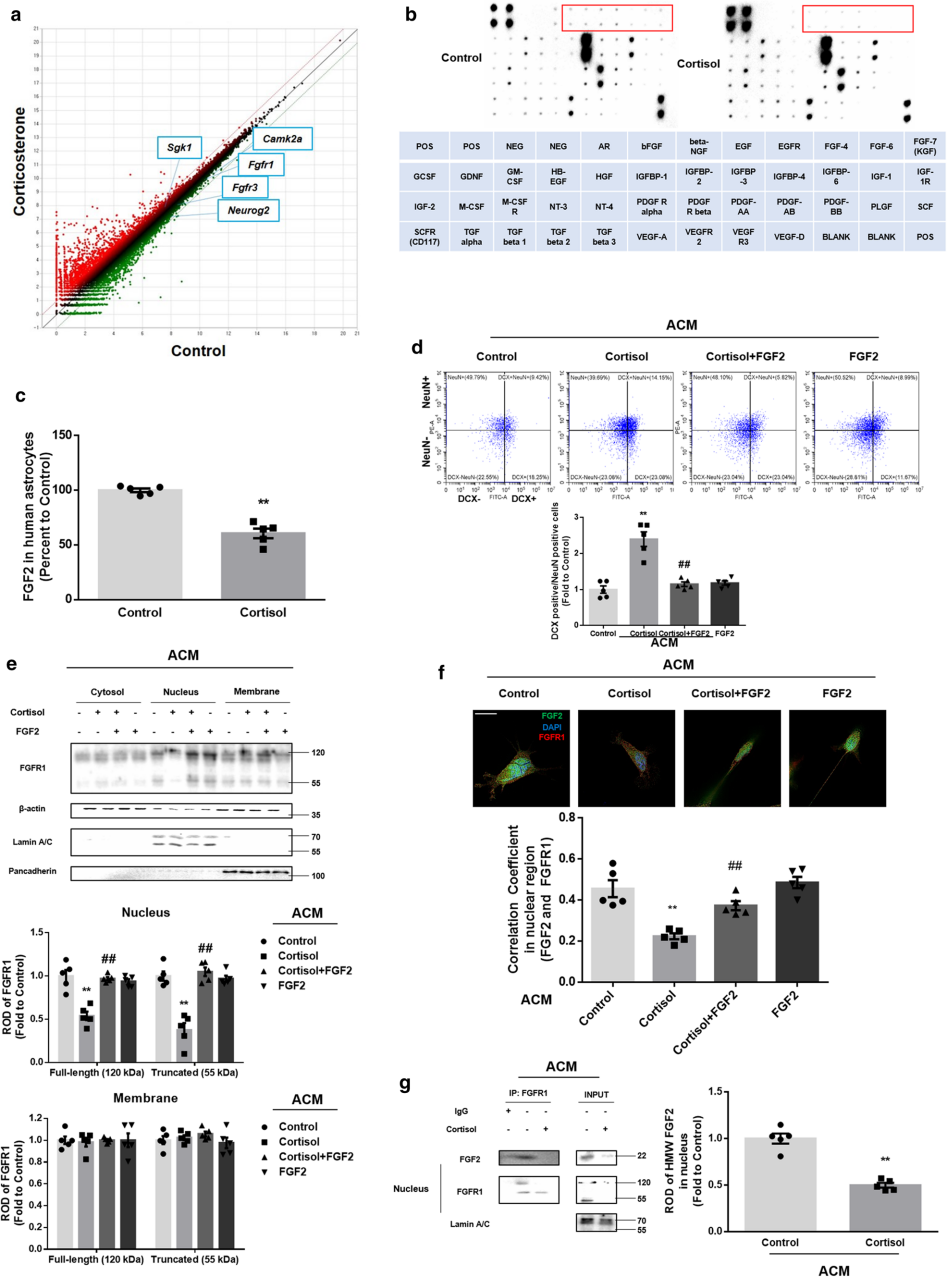
Glucocorticoid suppresses neurogenesis by reducing astrocytic HMW FGF2 and its downstream INFS. **a** After exposure to prenatal vehicle or corticosterone (10 mg/kg) at E14, the hippocampus from mice at P1 was collected. RNA sequencing was performed using RNA extracted from the hippocampal tissue. The scatter plot represents the log-fold changes between the mice exposed to prenatal vehicle (control) and the mice exposed to prenatal corticosterone. *n* = 3. (**b**, **c**) Five days after astrocytic differentiation, human NSCs were treated with cortisol (1 μM) for 48 h, and then the media were collected. **b** The growth factor antibody array was performed using the extracted media. *n* = 5. Red rectangles indicate significantly downregulated levels of growth factors between control and cortisol. The table indicates the location of detected factors in membrane. **c** Human FGF2 levels were measured using ELISA. FGF2 levels were decreased by cortisol-treated ACM. *n* = 5. (**d**–**g**) 5 days after astrocytic differentiation, human NSCs were treated with cortisol (1 μM) for 48 h and the ACM were collected. **d** The neurons differentiated from human NSCs (DIV 7) were treated with ACM and FGF2 (2 ng/ml) for 5 days. Then, the cells were immunostained with DCX (green) and NeuN (red). The ratio of DCX-positive cells to NeuN-positive cells was analyzed by flow cytometry. Upregulated ratio of DCX to NeuN-positive cells by cortisol-treated ACM was normalized by FGF2 treatment. *n* = 5. (**e**–**g**) The neurons (DIV 7) were treated with ACM and FGF2 for 24 h as appropriate. (**e**) Protein levels of FGFR1 in subcellular fraction samples were detected by western blotting. The β-actin was used as a loading control for cytosol. The lamin A/C and pancadherin were used as a loading control for nucleus and membrane, respectively. Only nuclear FGFR1 levels were downregulated by cortisol-treated ACM, recovered by FGF2. *n* = 5. **f** The cells were immunostained with FGF2 (green), FGFR1 (red), and DAPI (blue). Pearson’s correlation coefficient between FGF2 and FGFR1 in nuclear region was quantified. Cortisol-treated ACM reduced correlation coefficient value, recovered by FGF2. Scale bars, 20 μm (magnification × 1000). *n* = 5. **g** Subcellular fraction was done and FGFR1 was co-immunoprecipitated with FGF2 in nuclear parts. HMW FGF2 levels in immunoprecipitated samples were quantified, which were reduced by cortisol-treated ACM. *n* = 5. All immunofluorescence images are representative. *n* = 5 from independent experiments with two technical replicates each. Quantitative data are presented as a mean ± S.E.M. The representative images were acquired by SRRF imaging system. Two-sided unpaired student’s *t* test (Fig. 4c, g). Two-sided two-way ANOVA (Fig. 4d, e, f). ^**^indicates *p* < *0.01* versus control and ^##^indicates *p* < *0.01* versus cortisol, respectively

Based on the observation that cortisol-treated ACM impaired synaptic trafficking of GluR1/2, we performed RNA sequencing to get an insight into which neurotrophic factor-dependent signaling pathway represses glutamatergic transmission during neurogenesis in mice exposed to prenatal corticosterone. We compared the gene expression profiles of control mice and those exposed prenatally to corticosterone at E14. Scatter plots revealed that the mRNA expression of several neurotrophic factor-associated genes including *Fgfr1, Fgfr3*, and *Camk2a* was reduced more than two-fold following corticosterone treatment (Fig. 4a). Gene ontology analysis was also shown in supplementary Fig. 2a. Given that astrocytes secrete a variety of growth factors in support of neurogenesis, we determined the levels of neurotrophic factors released by astrocytes. Performing a growth factor antibody array, cortisol particularly decreased astrocytic fibroblast growth factors (Fig. 4b). These results led us to assume that FGF2 is key to maintaining synaptogenesis and subsequent neurogenesis during early life in mice since FGFR is highly associated with FGF2. FGF2 levels were decreased in extracted media from astrocytes (DIV 7) under cortisol treatment (Fig. 4c). Consistently, FGF2 levels in both mouse hippocampal tissue exposed to prenatal corticosterone and media from mouse primary hippocampal astrocytes (DIV 7) were reduced (Supplementary Fig. 2b, c). However, the FGF2 levels released from human differentiating neurons (DIV 7) were not significantly changed by cortisol, indicating that astrocytes play an important role in FGF2 support (Supplementary Fig. 2d). Furthermore, FGF2 levels in fully differentiated astrocytes (DIV 21) following 48 h of cortisol treatment were not significantly changed (Supplementary Fig. 2e). These results suggested that early-stage astrocytes play a key role in FGF2 release. We investigated whether pretreatment with human recombinant FGF2 rescues the detrimental effects of cortisol-treated ACM on neurogenesis and found that FGF2 recovered dysregulated neurogenesis by restoring the ratio of DCX-positive to NeuN-positive cells (Fig. 4d). As shown in (Fig. 4a), FGFR1-mediated signaling may be suppressed by astrocytic FGF2 reduction. FGFR1s are localized either in the cytosol, nucleus, or membrane, each of which has a distinct signaling pathway [34]. To differentiate which signaling pathway is activated, we performed subcellular fraction and immunostaining to find that cortisol-treated ACM selectively decreased the levels of nuclear FGFR1, which were recovered by FGF2 pretreatment (Fig. 4e). Similarly, the immunostaining results showed that the amount of FGF2–FGFR1 complex in the nucleus was reduced by cortisol-treated ACM, and this was reversed by FGF2 pretreatment (Fig. 4f). FGF2 is classified into high molecular weight (HMW 22–25 kDa) and low molecular weight (LMW 18 kDa) isoforms. FGFR1 bound to LMW FGF2 at the membrane usually acts as a receptor tyrosine kinase (RTK) and normally activates other kinases responsible for mitogenic function. As it contains the nuclear localization sequence, HMW FGF2 and its receptor FGFR1 were transported into the nucleus. When trafficking into nucleus, large parts of full-length FGFR1 (120 kDa) become truncated (55–60 kDa) and both forms bound to HMW FGF2 normally promote neural differentiation by binding to DNA [34, 35]. This pathway is called integrative nuclear FGFR1 signaling (INFS), because nuclear FGFR1 cooperates with the CREB-binding protein and many other transcription factors such as RXR, CREB, and estrogen receptor [36–38]. To confirm whether the interaction between HMW FGF2 and INFS was selectively affected, we performed immunoprecipitation assays of nuclear parts. Cortisol-treated ACM selectively suppressed nuclear FGFR1 binding to HMW FGF2 rather than LMW FGF2, indicating that neuronal INFS was suppressed by cortisol-treated ACM (Fig. 4g).

**Fig. 5:**
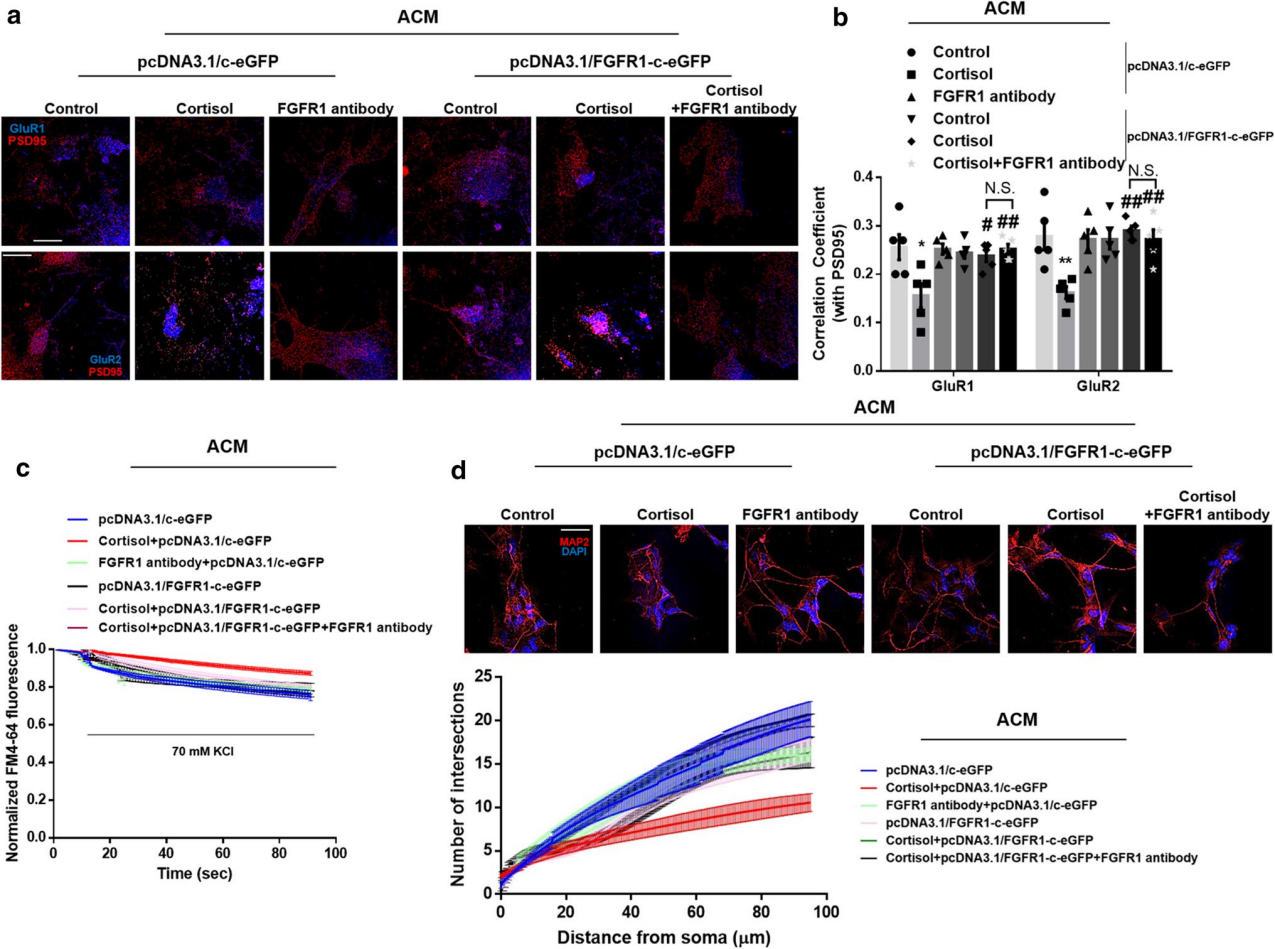
Reduction in INFS by cortisol inhibits AMPA-mediated glutamatergic synaptogenesis. (**a**–**d**) 5 days after astrocytic differentiation, human NSCs were treated with cortisol (1 μM) for 48 h and the ACM were collected. The neurons differentiated from human NSCs (DIV 6) were transfected with pcDNA3.1/c-eGFP or pcDNA3.1/FGFR1-c-eGFP for 24 h prior to ACM treatment for 48 h. Then, FGFR1 antibody (100 μg/ml) was pretreated for 1 h prior to ACM treatment for 48 h. (**a**, **b**) The cells were immunostained with GluR1/2 (blue) and PSD95 (red). Pearson’s correlation coefficient between GluR1/2 and PSD95 was quantified. Decreased co-localization by cortisol-treated ACM was attenuated by overexpression of *FGFR1* but FGFR1 antibody did not reverse these effect. Scale bars, 20 μm (magnification × 1000). *n* = 5. Two-sided two-way ANOVA was performed. **c** The neurons were stimulated with a high K^+^ buffer for destaining. Time-lapse imaging was performed over 90 s at 1 s intervals with an Eclipse Ts2™ fluorescence microscopy. The recovery effect of FGFR1 overexpression on reduced synaptic vesicle recycling by cortisol-treated ACM was not blocked by FGFR1 antibody. *n* = 5. **d** Conditioned neurons were immunostained with MAP2 (red) and DAPI (blue). The Sholl analysis was performed for analyzing neurite outgrowth. Reduced neurite outgrowth by cortisol-treated ACM was recovered by overexpression of *FGFR1*, which was not changed by the addition of FGFR1 antibody. Scale bars, 50 μm (magnification × 400). *n* = 5. All immunofluorescence images are representative. *n* = 5 from independent experiments with two technical replicates each. Quantitative data are presented as a mean ± S.E.M. The representative images were acquired by SRRF imaging system. ^*, **^indicates *p* < *0.05, p* < *0.01* versus control, respectively. ^*#, ##*^ indicates *p* < *0.05, p* < *0.01* versus cortisol, respectively

We then evaluated whether nuclear FGFR1 mainly participates in regulating glutamatergic synapse formation. We neutralized membrane FGFR1 with FGFR1 antibody to determine whether the adverse effects of cortisol-treated ACM depend on lower levels of nuclear FGFR1 in neurons. Trafficking of GluR1/2 into the postsynaptic compartment in differentiating neurons was blocked by cortisol-treated ACM but overexpression of *FGFR1* attenuated these effects. However, FGFR1 antibody did not reverse these recovery effects, suggesting that INFS plays an important role in maintaining the AMPA-mediated glutamatergic pathway (Fig. 5a, b). Furthermore, synaptic vesicle recycling recovered by FGFR1 upregulation was not blocked by the FGFR1 antibody (Fig. 5c). We also detected dendritic spine density to determine the amount of synaptogenesis. As shown in Fig. 5d, reduced neurite outgrowth caused by cortisol-treated ACM was recovered by overexpression of *FGFR1*, which remained unchanged by the addition of FGFR1 antibody. Altogether, decreased astrocytic FGF2 by glucocorticoid triggered defects in glutamatergic synaptogenesis, which was mainly dependent on a suppressed INFS.

### Neuroligin 1 downregulation by glucocorticoid reduced glutamatergic pathway-dependent neurogenesis and triggered behavioral deficits

**Fig. 6:**
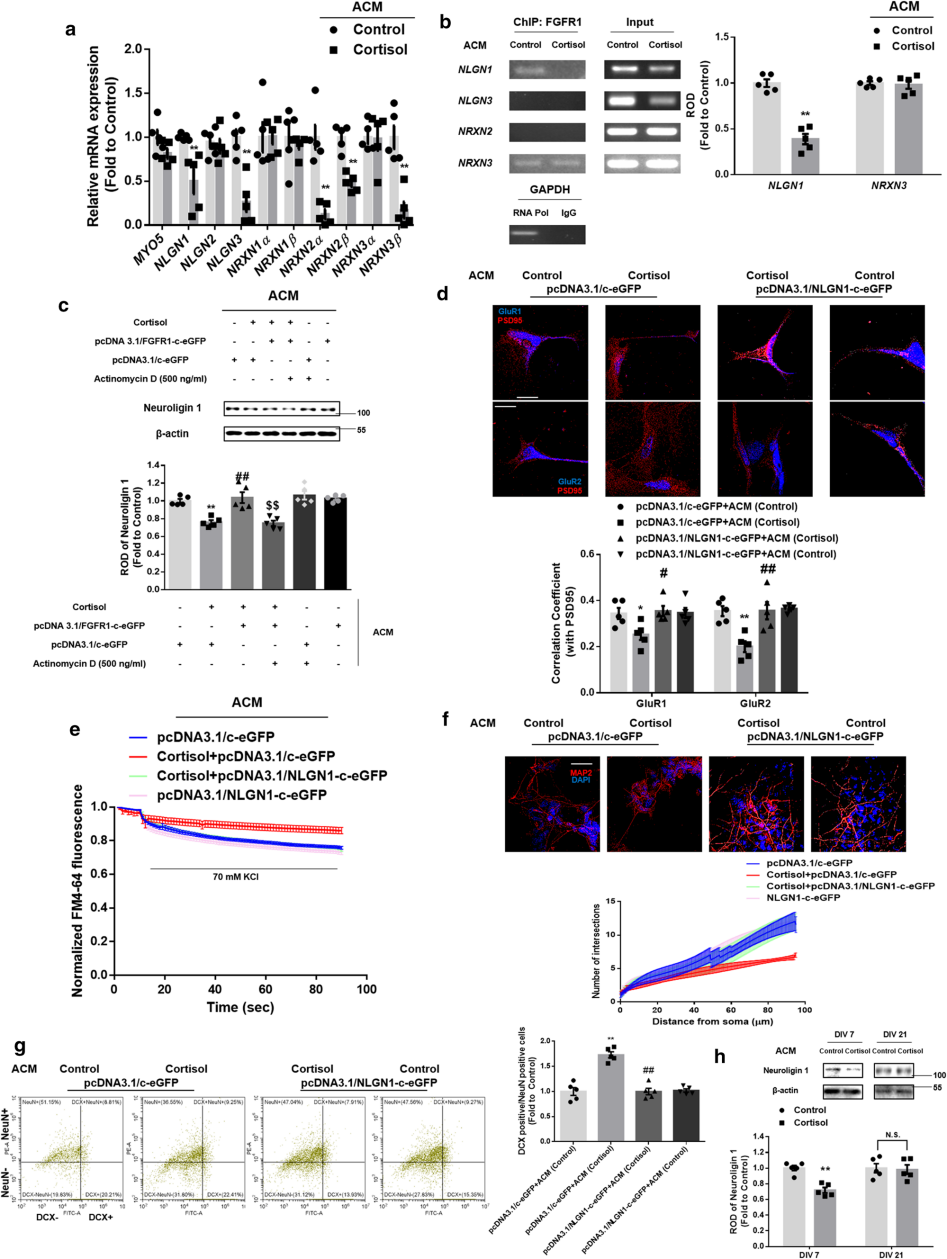
Suppressed INFS by cortisol downregulates neuroligin 1 and subsequent neurogenesis. (**a**–**h**) 5 days after astrocytic differentiation, human NSCs were treated with cortisol (1 μM) for 48 h and the ACM were collected. (**a**, **b**) The neurons differentiated from human NSCs (DIV 7) were incubated with ACM as appropriate for 24 h **a** The mRNA expressions were analyzed by real-time PCR. The genes for neuroligin 1/3, neurexin 2α/β, and neurexin 3β were downregulated by cortisol-treated ACM*. n* = 5. **b** DNA was immunoprecipitated with the IgG, RNA polymerase, and FGFR1 antibody. The samples were amplified with primers of *GAPDH*, *NLGN1*, *NLGN3*, *NRXN2*, and *NRXN3* genes. Relative optical density of gel bands was quantified by using image J. Cortisol-treated ACM reduced the binding of FGFR1 to the *NLGN1* promoter. *n* = 5. **c** The neurons differentiated from human NSCs (DIV 6) were transfected with pcDNA3.1/c-eGFP or pcDNA3.1/FGFR1-c-eGFP for 24 h prior to ACM treatment for 48 h. Actinomycin D (500 ng/ml) was pretreated for 30 min before ACM treatment as appropriate. Expression levels of neuroligin 1 were detected by western blotting. Overexpression of *FGFR1* recovered neuroligin 1 expression, but actinomycin D did not reverse the effect of cortisol-treated ACM on neuroligin 1 expression. ^$$^indicates*, p* < *0.01* versus cortisol with pcDNA3.1/FGFR1-c-eGFP transfection. *n* = 5. (**d**–**g**) The neurons differentiated from human NSCs (DIV 6) were transfected with pcDNA3.1/c-eGFP or pcDNA3.1/NLGN1-c-eGFP for 24 h prior to ACM treatment. (**d**–**f**) The ACM were administered to conditioned neurons for 48 h. **d** The neurons were immunostained with GluR1/2 (blue) and PSD95 (red). Pearson’s correlation coefficient between GluR1/2 and PSD95 was quantified. Overexpression of *NLGN1* recovered GluR1/2 trafficking into the postsynaptic compartment. Scale bars, 20 μm (magnification × 1000). *n* = 5. **e** The neurons were stimulated with a high K^+^ buffer for destaining. Time-lapse imaging was performed over 90 s at 1 s intervals with an Eclipse Ts2™ fluorescence microscopy. Recycled synaptic vesicles were normalized by *NLGN1* overexpression. *n* = 5. **f** The neurons were immunostained with MAP2 (red) and DAPI (blue). Sholl analysis was performed for analyzing neurite outgrowth. Overexpression of *NLGN1* recovered neurite outgrowth, which was suppressed by cortisol-treated ACM. Scale bars, 50 μm (magnification × 400). *n* = 5. **g** ACM were administered to the conditioned neurons for 5 days. The neurons were then immunostained with DCX (green) and NeuN (red). The ratio of DCX-positive cells to NeuN-positive cells was analyzed by flow cytometry. Overexpression of *NLGN1* recovered the decreased neurogenesis by cortisol-treated ACM. *n* = 5. **h** The differentiated neurons (DIV 7 or 21) were treated with ACM as appropriate. Neuroligin 1 expressions were detected by western blotting. Neuroligin 1 levels in differentiating neurons (DIV 7) were reduced by cortisol-treated ACM. *n* = 5. All immunofluorescence images are representative. *n* = 5 from independent experiments with two technical replicates each. Quantitative data are presented as a mean ± S.E.M. The representative images were acquired by SRRF imaging system. Two-sided unpaired student’s *t* test was conducted in (Fig. 6a, b, h). Two-sided two-way ANOVA was conducted in (Fig. 6c, d, g). ^*, **^indicates *p* < *0.05, p* < *0.01* versus control, respectively. ^#, ##^indicates *p* < *0.05, p* < *0.01* versus cortisol, respectively

Nuclear FGFR1 bound to HMW FGF2 attaches to DNA and acts as a transcription factor for various neurogenesis and neural activity-associated genes [34]. Because changes in GluR1/2 expression were not detected in our results, we assumed that synaptic GluR1/2 was selectively reduced by suppressed INFS. To maintain the synaptic pools of GluR1/2, transporting or scaffolding GluR1/2 into the postsynaptic compartment is critical to glutamatergic synapse formation and subsequent long-term potentiation (LTP). Thus, we observed mRNA expression of *MYO5*, which transports GluR1/2 into the synapse, and synaptic cell adhesion-associated genes, including *NLGN* and *NRXN* isoforms. The genes for neuroligin 1/3, neurexin 2α/β, and neurexin 3β were downregulated by cortisol-treated ACM (Fig. 6a). Nuclear FGFR1-targeted motifs are known to include a core AGGTCA sequence and we searched for this putative region for FGFR1 binding to the promoters of downregulated genes [35]. Thus, we performed in silico assay and designed the primers referring to the results (Supplementary Fig. 3a–d). Then, ChIP was performed to determine the genes most highly regulated by nuclear FGFR1. As shown in Fig. 6b, cortisol-treated ACM only reduced the binding of FGFR1 to the *NLGN1* promoter, while binding to the *NRXN2/NLGN3* promoter was not observed and that to the *NRXN3* promoter remained the same. Overexpression of *FGFR1* also recovered neuroligin 1 expression, but the transcription inhibitor actinomycin D did not reverse the effect of cortisol-treated ACM on neuroligin 1 expression, indicating that INFS plays a pivotal role in neuroligin 1 transcription (Fig. 6c). Furthermore, overexpression of *NLGN1* recovered GluR1/2 trafficking into the postsynaptic compartment, synaptic vesicle recycling, neurite outgrowth, and neurogenesis (Fig. 6d–g). We tested whether neuroligin 1 was altered in fully differentiated neurons to confirm that cortisol-treated ACM critically affects early neurogenesis of NSCs. Neuroligin 1 levels in fully differentiated neurons (DIV 21) was not changed by exposure to cortisol-treated ACM, unlike those in differentiating neurons (DIV 7) (Fig. 6h). Furthermore, we even tested the effect of GR-mediated signaling in neuroligin 1 expression. Since GR expression can vary depending on the stages of neuronal differentiation, we tried to demonstrate that neuroligin 1 expression is highly associated with other signaling pathways independent from GR at the early stage. Knockdown of *GR* did not further decrease neuroligin 1 expression suppressed by cortisol-treated ACM (Supplementary Fig. 4). Collectively, these results demonstrated that neuroligin 1 downregulation caused by reduced INFS following exposure to cortisol plays an important role in impairing glutamatergic synapse formation and subsequent neurogenesis, especially in the early stage of differentiating NSCs.

**Fig. 7:**
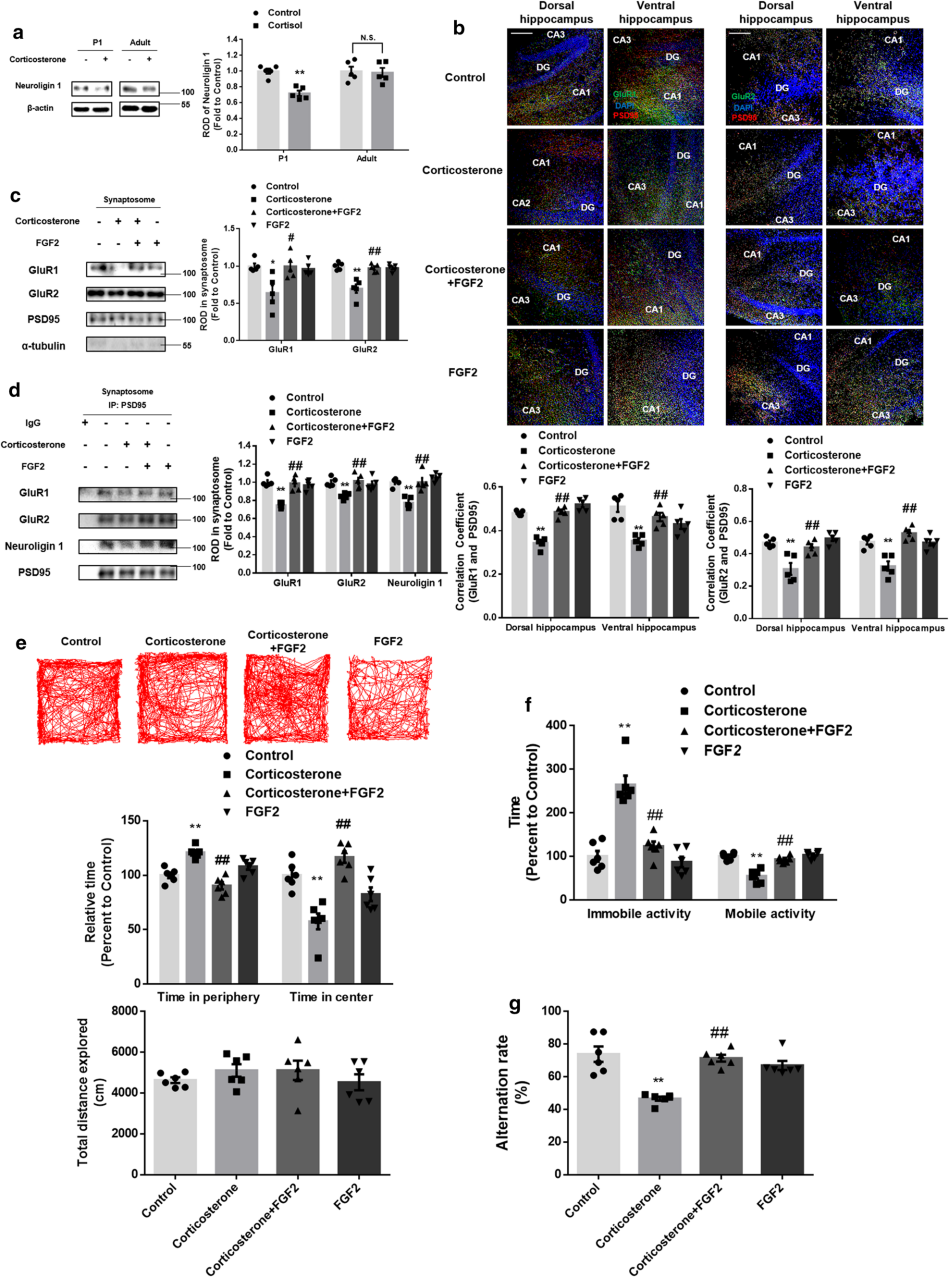
Restoration of neuroligin 1 by FGF2 pretreatment reverses the deleterious effects of corticosterone on glutamatergic synapse formation and hippocampal-related behaviors. **a** The E14 or adult mice (8 week-old) were exposed to corticosterone (10 mg/kg). 5 days after treatment, hippocampus was collected and neuroligin 1 was detected by western blotting of its lysates. Only prenatal corticosterone reduced neuroligin 1 expression. *n* = 5. (**b**–**g**) After exposure to maternal vehicle or corticosterone (10 mg/kg) at E14 and pretreatment of FGF2 starting at P1, P23 mice underwent behavior tests and were then sacrificed **b** Slide samples of both dorsal and ventral hippocampus for IHC were collected and hippocampal tissue was immunostained with GluR1/2 (green), PSD95 (red), and DAPI (blue). Pearson’s correlation coefficient between GluR1/2 and PSD95 was quantified. Reduced co-localization between GluR1/2 and PSD95 by corticosterone was reversed by FGF2 treatment. Scale bars, 100 μm (magnification × 200). *n* = 5. **c** The hippocampal tissue underwent subcellular fraction to gain synaptosome. The expressions of GluR1/2, PSD95, and α-tubulin were detected by western blot. PSD95 and α-tubulin were used as a loading control for synaptosome and cytosol, respectively. Decreased GluR1/2 levels in synaptosome were recovered by FGF2 pretreatment. *n* = 5. **d** PSD95 was co-immunoprecipitated with GluR1/2 and neuroligin 1 in synaptosome. Decreased GluR1/2 and neuroligin 1 levels in the postsynaptic compartment were recovered by FGF2 pretreatment. *n* = 5. **e** Open field test was performed. Relative time at the periphery/center and total distance explored were determined. FGF2 treatment drove mice to explore the center of the open field. *n* = 6. **f** Mice were presented to the forced swim test for 6 min. Immobile and mobile activities were determined during the last 4 min. FGF2 treatment rescued the depressive-like behavior induced by corticosterone exposure. *n* = 6. **g** The mice were subjected to Y-maze test to evaluate spatial memory function. Impaired spatial memory by corticosterone was attenuated by FGF2 treatment. *n* = 6. All immunofluorescence images are representative. Two technical replicates from each animal (*n* = 5) were performed in results of IHC or western blotting. Quantitative data are presented as a mean ± S.E.M. The representative images were acquired by SRRF imaging system. Two-sided two-way ANOVA was conducted except (Fig. 7a), data of which were analyzed by one-way ANOVA ^**^indicates *p* < *0.01* versus control and ^*##*^indicates *p* < *0.01* versus corticosterone

With the pathway having been established, we performed in vivo experiments to explore corticosterone-induced dysfunctions in brain. To confirm the significant role of prenatal glucocorticoid on neuroligin 1 expression, we administered corticosterone to mice at E14 and 8 week-old mice to compare the neuroligin 1 expression between the two groups. Similar to the in vitro results, fetal corticosterone exposure downregulated neuroligin 1, but no significant change in neuroligin 1 levels was seen in the adults (Fig. 7a). To assess glutamatergic synapse formation, we stained brain tissue with vGLUT1 and PSD95. Pretreatment with mouse FGF2 increased the interaction between vGLUT1 and PSD95, which was reduced by prenatal glucocorticoid exposure (Supplementary Fig. 5a). Furthermore, pretreatment with FGF2 normalized co-localization between GluR1/2 and PSD95, demonstrating that it attenuated the inhibitory effect of corticosterone on the postsynaptic trafficking of GluR1/2 (Fig. 7b). We also performed synaptosome isolation and observed that synaptic trafficking of GluR1/2 was recovered by FGF2 pretreatment despite corticosterone exposure (Fig. 7c). Consistently, an immunoprecipitation assay demonstrated that the reduced localization of GluR1/2 and neuroligin 1 in the postsynaptic compartments due to corticosterone treatment was restored by FGF2 pretreatment (Fig. 7d). Moreover, we showed that the reduction in nuclear FGFR1 caused by prenatal glucocorticoid exposure was reversed by FGF2 pretreatment (Supplementary Fig. 5b). Decreased glutamatergic synaptogenesis and neurogenesis in the hippocampus eventually culminate in behavioral changes such as anxiety-like behavior, major depressive disorder, and spatial memory dysfunction. We confirmed that FGF2 pretreatment drove mice to explore the center of the open field rather than the periphery (Fig. 7e). As shown in the results of forced swim test, FGF2 pretreatment rescued the depressive mood induced by corticosterone exposure (Fig. 7f). We also observed from the Y-maze alternation test results that corticosterone-treated mice exhibited impaired spatial memory, but pretreatment with FGF2 attenuated the damage to cognitive function (Fig. 7g). Altogether, neuroligin 1-dependent glutamatergic synapse formation and neurogenesis are selectively inhibited following prenatal exposure to corticosterone due to the suppression of the astrocytic FGF2–neuronal INFS axis.

## Discussion

**Fig. 8:**
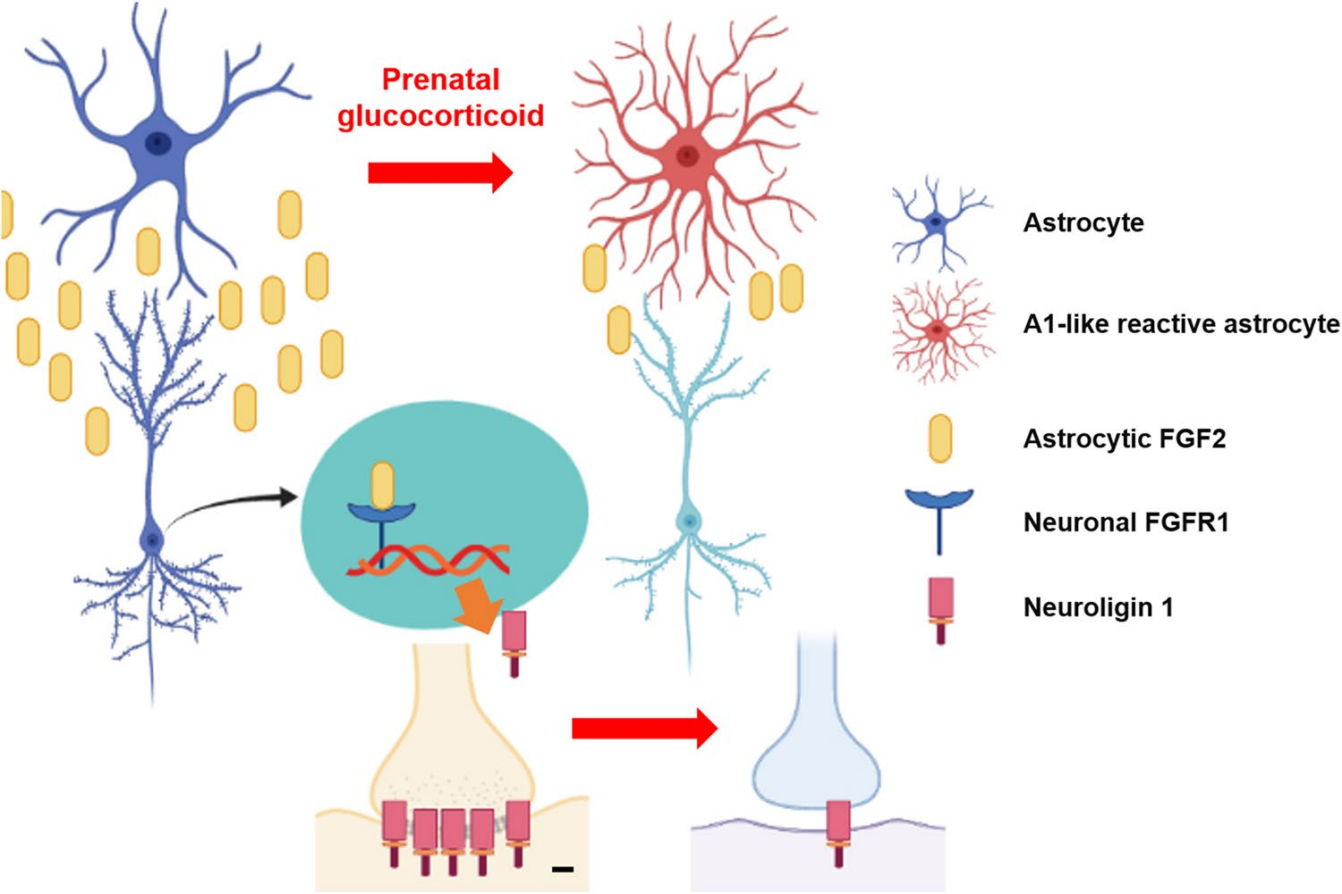
The schematic model for mechanisms of inhibition in neuroligin 1-dependent neurogenesis by prenatal glucocorticoid was shown. Astrocytic FGF2 and downstream nuclear neuronal FGFR1 trigger neuroligin 1 expression, which mainly causes glutamatergic synaptogenesis. However, prenatal glucocorticoid transforms astrocytes into A1-like reactive astrocytes and reduces FGF2 release, resulting in impaired synaptogenesis and subsequent neurogenesis

This study provides evidence that prenatal glucocorticoid exposure reduces neuroligin 1-dependent GluR1/2 trafficking into the synapse and subsequent neurogenesis in mouse hippocampus and in human iPSC-derived NSCs, and this decrease is induced by the downregulation of astrocytic FGF2 and the downstream INFS (Fig. 8). Our results and those from previous studies demonstrate that prenatal exposure to high levels of glucocorticoid affects both dorsal and ventral hippocampal neurogenesis, while this effect is not seen in infants or adults exposed to stress [2]. Sensitivity to stress is known to differ between the dorsal and ventral hippocampus [10]. For example, insignificant changes in spatial memory function in rodents exposed to early stress were observed although contradicting results have been found due to various early-life stress protocols [23, 39]. In contrast, our previous research demonstrated that only spatial memory dysfunction was detected while depressive behavior was not observed in adult mice following 1 week of exposure to corticosterone [21]. We speculated that these unique characteristics induced by prenatal corticosterone may be due to different epigenetic and transcriptome profiles between the two regions, which can create a discrepancy in the onset of spatial memory and mood disorders depending on the time of exposure to corticosterone [4, 10, 39, 40]. Furthermore, changes in GR levels during fetal exposure to prenatal stress can trigger unique cognitive or mood responses. Besides the fact that GR expression differs depending on the stage of neuronal differentiation, our results and previous studies have shown that prenatal corticosterone impairs the HPA axis in P23 mice, indicating that a reduction in GR would exaggerate HPA axis at this age [41]. Thus, other glucocorticoid-mediated crosstalk pathways independent from GR-mediated signaling such as changes in neurotrophic factors released from glial cells by prenatal glucocorticoid can largely affect differentiation in immature neurons [2, 8]. Finally, we presumed that the distinct characteristics of hippocampal neurogenesis during the prenatal period also contribute to the deleterious effect of glucocorticoid on neural differentiation. Prenatal stress drives NSC differentiation into glial cells, as in oligodendrogenesis, causing an inability to buffer stress due to insufficient pools of hippocampal NSCs or neurons [2, 7, 16]. The size of the NSC pool at the SGZ when responding to stress also contributes to hippocampal neurogenesis, because relatively large portions of infant NSCs contribute to brain circuit formation, unlike adult NSCs which are usually differentiated to buffer stress. Collectively, it is plausible that prenatal glucocorticoid predisposes animals and humans to develop cognitive or neuropsychiatric disorders through distinct mechanisms that negatively affect neurogenesis, unlike early-life or adult stress.

We have demonstrated that prenatal increase in glucocorticoid levels promotes astrocytes to become A1-like reactive astrocytes, which are toxic to synaptogenesis and neurogenesis due to their inability to release neurotrophic factors. We thereby investigated the research regarding the effect of ACM on synapse formation of NSC and found that cortisol-treated ACM affected only AMPA-mediated glutamatergic synapse formation. GABAergic and glutamatergic neurons represent two major neuronal classes, which establish inhibitory and excitatory synapses, respectively. In the SGZ, GABAergic interneurons regulate synaptic integration in early neurogenesis, while glutamatergic mechanisms maintain neuronal plasticity at later stages in neurogenesis [42, 43]. Our results and previous work suggest that prenatal glucocorticoid exposure dominantly influences the later stage of neurogenesis, which agrees with the finding that the migration of GABAergic interneurons does not require certain neurotrophic factors such as FGF2 [44]. We then determined why cortisol-treated ACM selectively amplifies the defects in AMPA-mediated glutamatergic synaptogenesis. To activate glutamatergic synapse formation, the ratio of GluR1/2 to NR in the synapse increases, because GluR subunits are major contributors to LTP formation. And incorporation of AMPAR into the excitatory synapse later activates NMDAR, which can form the late phase of LTP even weeks after the stimuli [1, 45–47]. In our research, prenatal glucocorticoid selectively decreased AMPA-mediated synaptic transmission, but only the synaptic trafficking of GluR subunits was suppressed, unlike the downregulation of GluR1 and NR1 shown in neurons of the adult rats given 4 weeks of repeated stress [9]. High levels of glucocorticoid are widely known to ultimately induce upregulations of NMDAR levels due to the elevated endocytosis of GluR subunits [33, 47, 48]. Thus, there is a possibility that longer term observations of LTP formation following prenatal corticosterone exposure would offer different results from our results; however, we presumed that discrepancies can occur due to the different neurotrophic factors that contribute to glutamatergic synapse formation depending on the period of glucocorticoid exposure.

Our transcriptomic approach and comparison depending on the timing of glucocorticoid exposure revealed that astrocytic FGF2 release was significantly reduced in the hippocampus under prenatal corticosterone and in differentiating astrocytes from human NSCs under cortisol treatment, unlike stress-exposed adult mice or fully differentiated astrocytes. In fetuses or neonates, profiles of and sensitivity to neurotrophic factors are different from those in adulthood. For example, FGF2 is abundant in embryonic brains compared to other neurotrophic factors and peaks at E14–E18 in rodents. It is maintained at a high level until the early-postnatal stage to conserve the astrocytic and neuronal pool but significantly decreases during mid-to-late neurogenesis [15, 44]. Thus, excessively low levels of FGF2 inhibit the proliferation and differentiation of astrocytes, so that NSC pools remain undifferentiated, decreasing neurogenesis and gliosis in early life [22, 23]. In contrast, FGF4, which is deficient in neonatal precursor cells, dominates during the later stages [14]. Other isotypes such as FGF3, FGF7, FGF8, FGF15, FGF17, and FGF18 regulate neurogenesis in specific regions, whereas FGF2 affects both neurogenesis and gliosis throughout the brain [49]. These findings suggest that the time-specific effects of FGF2 dominantly and selectively occur during early NPC activity when they are susceptible to prenatal stress. Interestingly, the levels of FGF2 released from differentiated neurons did not change significantly, which is consistent with previous research demonstrating that astrocytes are the dominant FGF2-producing cells in the CNS [50]. Collectively, it can be inferred that a decreased astrocytic pool and an elevated number of neurotoxic astrocytes caused by prenatal glucocorticoid worsen neurogenesis by reducing fetal or neonatal FGF2 release.

FGF2 is widely known to affect neurogenesis through its receptor FGFR. FGFR isotypes distribute differentially in the brain; FGFR1/4 is predominant in neurons whereas FGFR2/3 mainly exists in oligodendrocytes and astrocytes [22]. According to previous research, FGFR1 is highly expressed in NSCs and the FGF2–FGFR1 system is especially critical to CNS formation during early life, whereas FGFR4 is not essential to embryonic brain development [44]. Consistently, our results demonstrated that the decreased interaction between astrocytic FGF2 and neuronal FGFR1 due to prenatal glucocorticoid exposure is mostly responsible for suppressing glutamatergic synaptic transmission in NSCs. Importantly, however, our results are not consistent with the previous paradigm that the FGF2-FGFR1 system mainly activates the RTK action of FGFR1 by phosphorylating tyrosine 766, subsequently triggering downstream candidates such as PI3K, RAS-MAPK, and PKC [45, 51]. Previous research demonstrated that AMPAR-mediated synaptic transmission in neurons depends on the LMW FGF2-membrane FGFR1–PKC axis [22]. But it turned out that the interaction between HMW FGF2 and INFS is a key mediator of impaired glutamatergic synapse formation and the subsequent neurogenesis of NSCs, independently from RTK action triggered by surface FGFR1-mediated signaling. We must then ask why prenatal glucocorticoid exposure primarily affects nuclear FGFR1-dependent transcription in neurons. As mentioned above, signal transduction associated with neurotrophic factor-mediated neuronal differentiation occurs differentially in the various brain regions. For example, FGFR1 preferentially affects the ventral hippocampus, suggesting that the FGF2–FGFR1 system can be differentially activated [13]. Moreover, the different roles of FGFR1 which is present in intracellular compartments, or release of FGF2 isotypes can be changed depending on the type of stimuli [34]. FGFR1 is usually a membrane-directed protein, however, with HMW FGF2 stimulation, FGFR1 is released from the pre-Golgi membrane into the cytosol by Sec61 and the binding of ligands can change the number of FGFR1 molecules in each pool [34]. Furthermore, INFS is known to affect brain development and differentiation, unlike RTK action by membrane FGFR1 usually contributes to mitogenic function [35]. Thus, decreased HMW FGF2 release from astrocytes exposed to prenatal glucocorticoid would selectively suppress FGFR1 transcription. Another possibility we can also assume is that persistent changes in the epigenetic profiles derived from altered neurotrophic factors by prenatal glucocorticoid can affect the number of FGFR1s in each compartment and increase the nuclear pool facilitating DNA transcription in neurons [52]. The expression of molecules such as importin and notch that participate in FGFR1 translocation into the nucleus could be also differentially expressed, depending on the brain region or glucocorticoid exposure period; however, further studies are necessary [34]. Even though these speculations require further investigation, this is the first observation of the unique suppressive effects of prenatal glucocorticoid exposure glutamatergic synapse formation and neurogenesis by impairing the interaction between astrocytic FGF2 and neuronal INFS.

An important finding of this study is that decreased INFS caused by prenatal glucocorticoid exposure downregulated neuroligin 1, a critical mediator of glutamatergic synapse formation. Glutamate receptor-mediated synaptic transmission is mainly induced by an increased surface ratio of AMPAR/NMDAR, and the activation of an AMPA-mediated synaptic current subsequently activates various factors such as CaMKII, PKC, PKA, ERK, and CREB which aid neurogenesis and memory formation [33]. Among the genes participating in trafficking or scaffolding of GluR1/2 into synapse, we found that the downregulation of FGFR1 by cortisol-treated ACM, which can bind to various gene promoters, reduced its capacity to bind to *NLGN1* promoters, despite the presence of an FGFR1 binding to the *NRXN3* promoter. Neuroligin is a synaptogenic transmembrane protein that recruits glutamate/GABA receptors for postsynaptic potentiation and consists of four subtypes; neuroligin 1 is usually localized in excitatory synapses whereas neuroligin 2/4 are at inhibitory synapses, and neuroligin 3 exists in both types of synapse. Specifically, neuroligin 1 prefers AMPAR to NMDAR and scaffolds GluR1/2 into PSD95, subsequently forming an excitatory synapse [53]. Altogether, the present study supports the suggestion that the inhibitory effect of cortisol-treated ACM on neuroligin 1 expression selectively inhibits glutamatergic synaptogenesis by reducing the recruitment of GluR1/2 into the synapse. However, many open questions about the particular effect of prenatal stress on neuroligin 1 remain. During early life, FGF2 and INFS contribute critically to neurogenesis, since many other mechanisms such as GR-dependent signaling are activated differently at this stage. In contrast, prolonged severe stress in adult rodents reduces LTP through the genomic pathway, whereas acute stress increases synaptic transmission via nongenomic pathways, both of which we found to be insignificant [54]. These effects were due to the differential expression and distribution of GR in the neonatal brain because our results showed that knockdown of *GR* did not alter neuroligin 1 expression in differentiating neurons. Moreover, we could not observe significant changes in neuroligin 1 expression in fully differentiated neurons and adult mouse brains exposed to cortisol-treated ACM and corticosterone, respectively. These findings are supported by several reports comparing the effects of stress, depending on age. Effects of stress may vary depending on epigenetic factors such as chromatin structure and formation of topologically associating domains. These factors can change during cell development and the gene expression regulated by nuclear FGFR1 can thereby be different in responding to the timing of stress exposure [55]. Thus, it has been observed that chronic restraint stress in adulthood reduced neuroligin 2 but not neuroligin 1 expression in the hippocampus [56]. Another report also demonstrated that upregulation of neuroligin 1 did not induce synapse formation in adult-born dentate granule cells of the hippocampus [57]. The fact that neuroligin 1 is the most abundant isotype in the hippocampus also allows developing neurons from NSCs of the SGZ to rely heavily on it [58]. Collectively, these results support a unique mechanism susceptible to prenatal stress exists; in this present study, astrocytic FGF2-neuronal INFS axis that regulates neuroligin 1. Our in vivo data demonstrated that FGF2 restoration recover neuroligin 1-dependent glutamatergic synaptogenesis, inhibiting the progression of spatial memory loss, anxiety, and depressive mood. However, this approach neglects the potential or a systemic effect of FGF2 injection, which does not occur by overexpression of FGF2 in hippocampal astrocytes using AAV vectors; nevertheless, demonstrating the unique effects of prenatal corticosterone exposure on neuroligin 1 during early life can compensate for these limitations. In conclusion, to the best of our knowledge, our study is the first to identify neuroligin 1 and its regulator INFS as potential targets for treating prenatal glucocorticoid-induced neurogenesis defects and memory/mood disorders.

## Supplementary Information

Below is the link to the electronic supplementary material.

Supplementary file1 (PDF 1271 KB)

## Data Availability

The authors declare that all the data supporting the findings of this study are available within this article, its supplementary information files, or are available from the corresponding author, who has all relevant data, upon reasonable request. Source data including raw data are provided in ‘https://drive.google.com/drive/folders/15GKcdXCYov_SCtusLKg6XoyetWaJF4e3?usp=sharing’.

## Abbreviations

ACM: Astrocytic conditioned media
ALDH1L1: Aldehyde dehydrogenase 1 family member L1
AMPA: α-Amino-3-hydroxy-5-methyl-4-isoxazole propionic acid
AMPAR: AMPA receptor
ChIP: Chromatin immunoprecipitation
DCX: Doublecortin
E: Embryonic day
FGF2: Fibroblast growth factor 2
FGFR1: Fibroblast growth factor receptor 1
GR: Glucocorticoid receptor
GluR: Ionotropic glutamate receptor
HPA: Hypothalamus–pituitary–adrenal
INFS: Integrative nuclear FGFR1 signaling
iPSC: Induced pluripotent stem cell
LTP: Long-term potentiation
NGS: Normal goat serum
NMDA: N-Methyl-D-aspartate
NMDAR: NMDA receptor
NPC: Neuroprogenitor cell
NSC: Neural stem cell
NT: Nontargeting
P: Postnatal day
PI: Propidium iodide
RNAPol: RNA polymerase
RTK: Receptor tyrosine kinase
S.E.M.: Standard error of mean
SGZ: Subgranular zone
siRNA: Small interfering RNA
SRRF: Super-resolution radial fluctuations
