# Supplementary material for: Prenatal glucocorticoid exposure selectively impairs neuroligin 1-dependent neurogenesis by suppressing astrocytic FGF2–neuronal FGFR1 axis
Source: Cell Mol Life Sci. 2022 May 13;79(6):294. doi: 10.1007/s00018-022-04313-2 (PMC9106608; doi:10.1007/s00018-022-04313-2)
Supplement: Supplementary file 1 — Supplementary file1 (PDF 1271 KB) [file 18_2022_4313_MOESM1_ESM.pdf]

**Supplementary Figure 1**

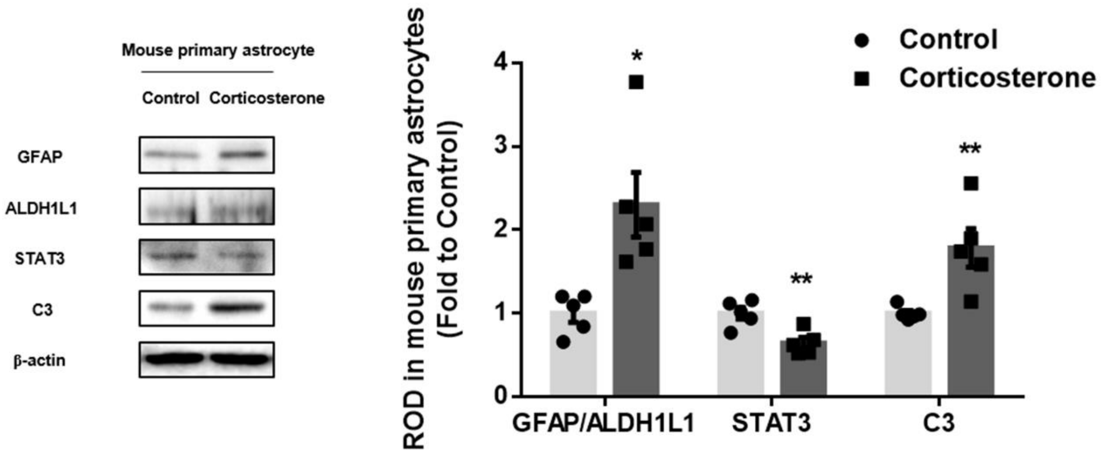


**Figure 1. Corticosterone shifts mouse hippocampal primary astrocytes into A1-like astrocytes.** Mouse hippocampal astrocytes (DIV 5) were treated with corticosterone (1 μM) for 48 h. The expressions of GFAP, ALDH1L1, STAT3, C3, and β-actin were detected by western blot. *n*=5. Quantitative data are presented as a mean ± S.E.M. $^{*,\ **}$ indicates *p<0.05, p<0.01* versus control, respectively.

**Supplementary Figure 2**

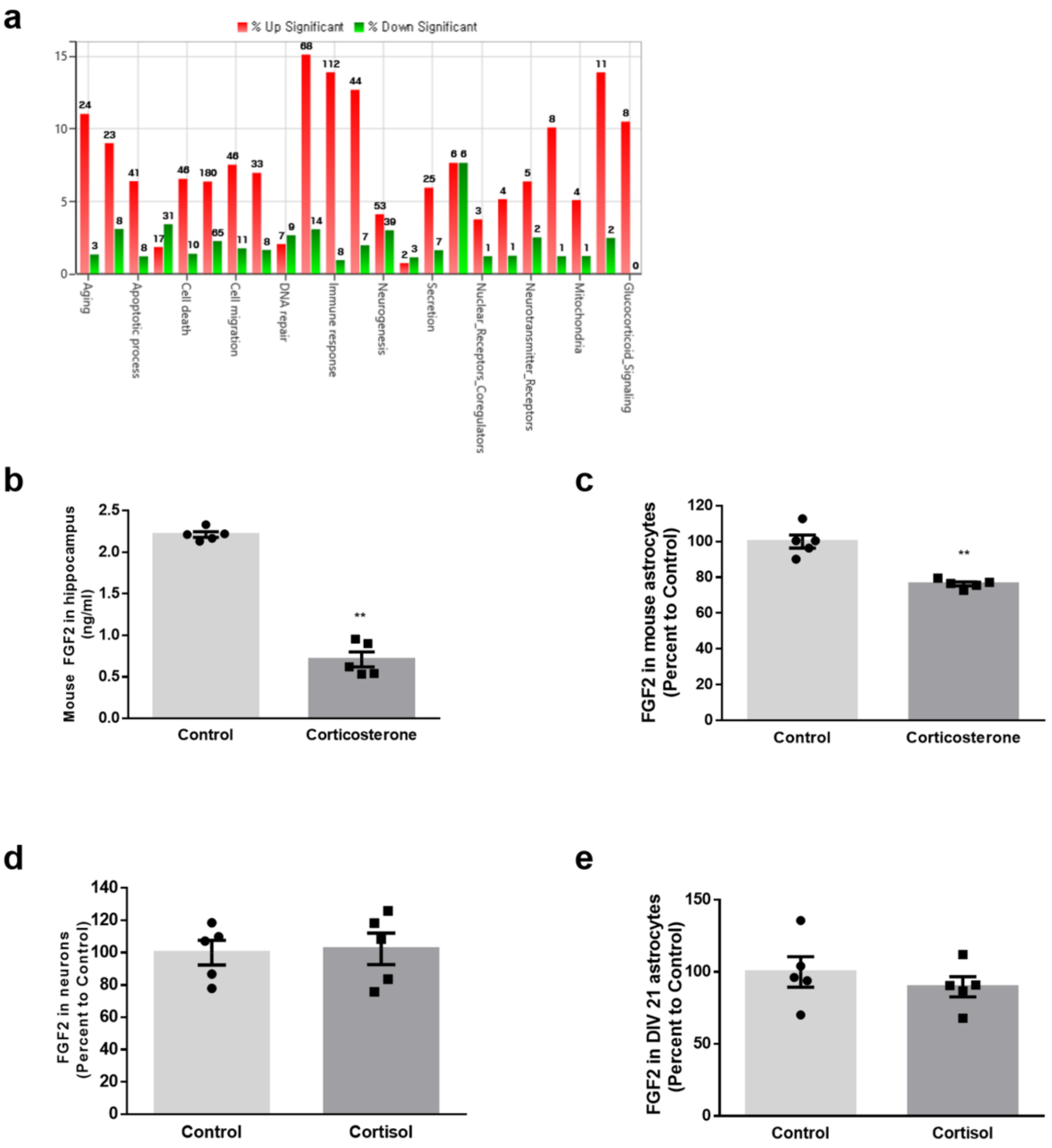

**Figure 2. Glucocorticoid only suppresses astrocytic FGF2 release at the early stage.** (**a-b**) After exposed to prenatal vehicle or corticosterone (10 mg/kg) at E14, the hippocampus from mice at P1 was collected. RNA sequencing was performed using RNA extracted from the hippocampal tissue. Gene ontology analysis was performed and the selected genes shown in the graph indicate the mRNA expression levels has changed more than twice the genes from control mice ($p$ value of < 0.05). $n$=3. (**b**) The tissue lysates were collected and mouse FGF2 levels were measured using ELISA. $n$=5. (**c**) Mouse hippocampal astrocytes (DIV 5) were treated with corticosterone (1 μM) for 48 h. Mouse FGF2 levels in cultured media were detected using ELISA. $n$=5. (**d**) Five days after neuronal differentiation of human NSCs, cortisol (1 μM) was treated for 48 h. Human FGF2 levels in the conditioned media were measured using ELISA. $n$=5. (**e**) Cortisol (1 μM) was treated for 48 h in astrocytes differentiated from human NSCs and media were harvested at DIV 21. Human FGF2 levels were detected by ELISA. $n$=5. Quantitative data are presented as a mean ± S.E.M. $^{**}$ indicates $p<0.01$ versus control.

**Supplementary Figure 3**

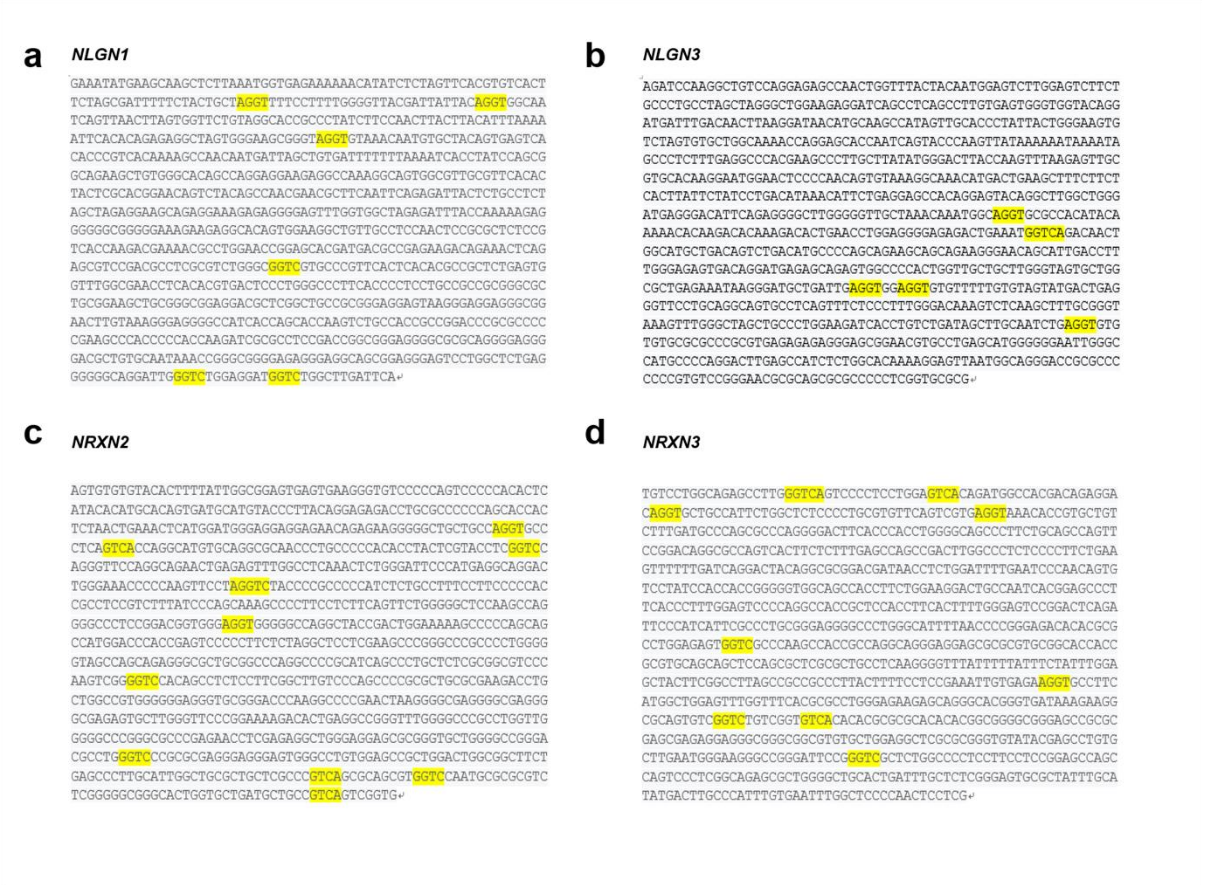


**Figure 3. Putative binding regions of FGFR1 in promoters of synaptic cell adhesion-associated genes. (a-d)** A thousand base pair upstream of the first codon of the *NLGN1*, *NLGN3*, *NRXN2*, and *NRXN3* was described and the putative FGFR1 binding sequence was emphasized with yellow labeling.

**Supplementary Figure 4**

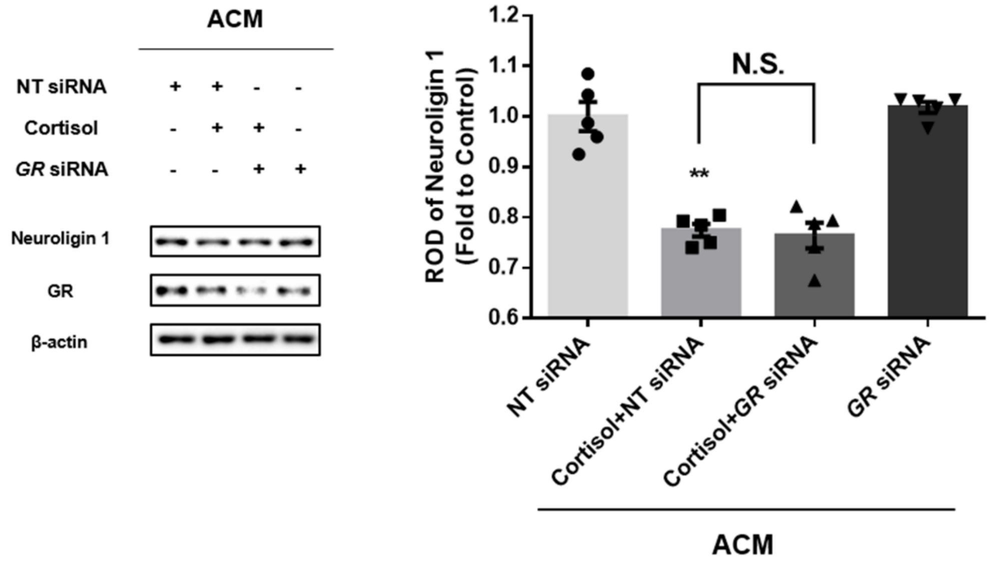


**Figure 4. Downregulation of neuroligin 1 by cortisol-treated ACM is independent from GR-mediated signaling.** Five days after astrocytic differentiation, human NSCs were treated with cortisol (1 μM) for 48 h and ACM were collected. The neurons differentiated from human NSCs (DIV 6) were transfected with *GR* siRNA for 24 h prior to ACM for 48 h. Expression of neuroligin 1 and GR was detected by western blotting. The β-actin was used as a loading control. *n*=5 from independent experiments with two technical replicates each. Quantitative data are presented as a mean ± S.E.M. ** indicates $p<0.01$ versus control.

**Supplementary Figure 5**

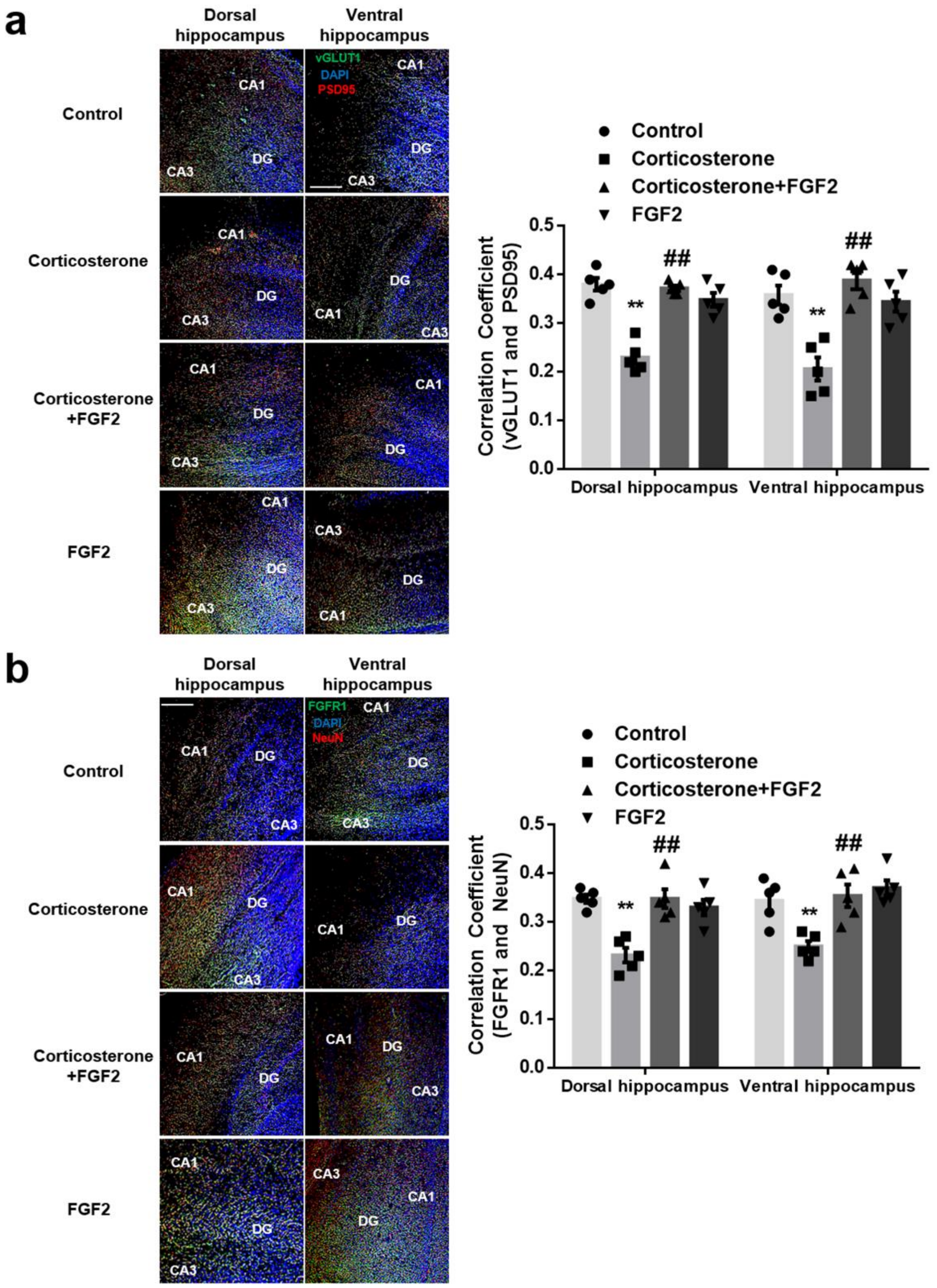

**Figure 5. Neuroligin 1 restoration by FGF2 protects hippocampal neurons from glutamatergic synaptogenesis defects.** (**a-b**) After exposure to maternal vehicle or corticosterone (10 mg/kg) at E14 and pretreatment of FGF2 starting at P1, P23 mice were sacrificed. (**a**) Slide samples of both dorsal and ventral hippocampus for IHC were collected and hippocampal tissue was immunostained with vGLUT1 (green), PSD95 (red), and DAPI (blue). Pearson's correlation coefficient between vGLUT1 and PSD95 was quantified. Scale bars, 100 μm (magnification, × 200). *n*=5. (**b**) Slide samples of both dorsal and ventral hippocampus for IHC were collected and hippocampal tissue was immunostained with FGFR1 (green), NeuN (red), and DAPI (blue). Pearson's correlation coefficient between FGFR1 and NeuN was quantified. Scale bars, 100 μm (magnification, × 200). *n*=5. All immunofluorescence images are representative. Two technical replicates per mouse (*n*=5) were performed in results of IHC or western blotting. Quantitative data are presented as a mean ± S.E.M. The representative images were acquired by SRRF imaging system. $^{**}$ indicates $p<0.01$ versus control and $^{\#\#}$ indicates $p<0.01$ versus corticosterone.
